# Exploration of the Antimicrobial Effects of Benzothiazolylthiazolidin-4-One and In Silico Mechanistic Investigation

**DOI:** 10.3390/molecules26134061

**Published:** 2021-07-02

**Authors:** Michelyne Haroun, Christophe Tratrat, Anthi Petrou, Athina Geronikaki, Marija Ivanov, Ana Ćirić, Marina Soković, Sreeharsha Nagaraja, Katharigatta Narayanaswamy Venugopala, Anroop Balachandran Nair, Heba S. Elsewedy, Hafedh Kochkar

**Affiliations:** 1 Department of Pharmaceutical Sciences, College of Clinical Pharmacy, King Faisal University, Al-Ahsa 31982, Saudi Arabia; christophetratrat@gmail.com (C.T.); sharsha@kfu.edu.sa (S.N.); kvenugopala@kfu.edu.sa (K.N.V.); Anair@kfu.edu.sa (A.B.N.); helsewedy@kfu.edu.sa (H.S.E.); 2 School of Pharmacy, Aristotle University of Thessaloniki, 54124 Thessaloniki, Greece; anthi.petrou.thessaloniki1@gmail.com; 3 Mycological Laboratory, Department of Plant Physiology, Institute for Biological Research, Siniša Stanković-National Institute of Republic of Serbia, University of Belgrade, Bulevar Despota Stefana 142, 11000 Belgrade, Serbia; marija.smiljkovic@ibiss.bg.ac.rs (M.I.); rancic@ibiss.bg.ac.rs (A.Ć.); mris@ibiss.bg.ac.rs (M.S.); 4 Department of Pharmaceutics, Vidya Siri College of Pharmacy, Off Sarjapura Road, Bengaluru 560 035, Karnataka, India; 5 Department of Biotechnology and Food Technology, Faculty of Applied Sciences, Durban University of Technology, Durban 4001, South Africa; 6 Department of Chemistry, College of Science, Imam Abdulrahman Bin Faisal University, Dammam 31441, Saudi Arabia; hbkochkar@iau.edu.sa; 7 Basic & Applied Scientific Research Center, Imam Abdulrahman Bin Faisal University, Dammam 31441, Saudi Arabia

**Keywords:** thiazolidinones, PASS, antibacterial, MIC/MFC, docking, LD-carboxypeptidase

## Abstract

Background: Infectious diseases still affect large populations causing significant morbidity and mortality. Bacterial and fungal infections for centuries were the main factors of death and disability of millions of humans. Despite the progress in the control of infectious diseases, the appearance of resistance of microbes to existing drugs creates the need for the development of new effective antimicrobial agents. In an attempt to improve the antibacterial activity of previously synthesized compounds modifications to their structures were performed. Methods: Nineteen thiazolidinone derivatives with 6-Cl, 4-OMe, 6-CN, 6-adamantan, 4-Me, 6-adamantan substituents at benzothiazole ring were synthesized and evaluated against panel of four bacterial strains *S. aureus*, *L. monocytogenes*, *E. coli* and *S. typhimirium* and three resistant strains MRSA, *E. coli* and *P. aeruginosa* in order to improve activity of previously evaluated 6-OCF_3_-benzothiazole-based thiazolidinones. The evaluation of minimum inhibitory and minimum bactericidal concentration was determined by microdilution method. As reference compounds ampicillin and streptomycin were used. Results: All compounds showed antibacterial activity with MIC in range of 0.12–0.75 mg/mL and MBC at 0.25–>1.00 mg/mL The most active compound among all tested appeared to be compound **18,** with MIC at 0.10 mg/mL and MBC at 0.12 mg/mL against *P. aeruginosa.* as well as against resistant strain *P. aeruginosa* with MIC at 0.06 mg/mL and MBC at 0.12 mg/mL almost equipotent with streptomycin and better than ampicillin. Docking studies predicted that the inhibition of LD-carboxypeptidase is probably the possible mechanism of antibacterial activity of tested compounds. Conclusion: The best improvement of antibacterial activity after modifications was achieved by replacement of 6-OCF_3_ substituent in benzothiazole moiety by 6-Cl against *S. aureus*, *MRSA* and resistant strain of *E. coli* by 2.5 folds, while against *L. monocytogenes* and *S. typhimirium* from 4 to 5 folds.

## 1. Introduction

Amalgamations of drugs endowed with different medicinal activities have been dispensed to patients for decades. It is known that an adapted adjustment of different targets may offer an improved medicinal aspect and an advantageous side effect in contrast with the effect of a ligand that has a unique mode of action [1]. In comparison with drug combinations, there are several betterments emanating from drugs active on several receptors, including the more foreseeable pharmacokinetics and pharmacodynamics properties resulting of the treatment of a unique medicinal drug in addition to ameliorated patient acquiescence.

Characterization of new therapies for both antifungal and antibacterial disorders that can operate more efficaciously and that are exempt of the side effects related to the present medications continues to be a principal requirement in pharmaceutical research [2,3]. Applying several drugs to infective symptoms in correlation with inflammation is an impasse, particularly when patients suffer from defective hepatic or renal failure or in countering interaction between two drugs [4]. Furthermore, from the standpoint of medical financial efficiency, and pursuing a favorable patient safety, a dual antifungal/antibacterial drug having the slightest side effects in addition to improved safeness profile must be energetically advisable [5].

The initial step in investigating for dual-acting molecules is constituted by screening collections of drugs to design pharmacophores’ coupling [6]. This investigation may be accomplished in vitro and in silico. It ultimately grasps effective compounds on appropriate enzymes belonging to identical or similar enzyme families. Analysis of the framework of ligands effective on different targets or enzymes together with protein similarity study, dimensions, and aspects of their effective cores may be adopted to find possible targets for dual-acting drugs [7,8].

**Figure 1:**
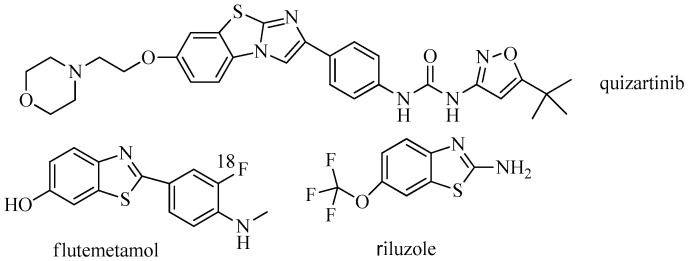
Drugs approved by FDA.

Computer-aided prediction of pharmacological activity spectra of compounds and drugs based on their structural formulas can be appraised by software Prediction of Activity Spectra for substances (PASS) [9] to perform investigations on novel antimicrobial. This strategy is built on the structure–activity relationship study in a heterogeneous data set. The set includes 989,000 various chemical compound families endowed with tremendous variety of biological potency. For the selection of compounds with predicted designed properties, PharmaExpert4 software was promoted. The version [10,11] is established from the literature data and furnished a single feasibility to look for compounds with potential multi-targeted activity. This study aims at discovering compounds endowed with anti-infective potency. It is worth mentioning that benzothiazole is a privileged heterocyclic scaffold with multiple applications and tremendous range of pharmacological activity. Benzothiazoles are recognized for their anti-inflammatory [12,13,14], antimicrobial [15,16,17,18,19], anesthetic [20], anticancer [21,22,23], anti-viral [24,25,26], analgesic [13,14], antipyretic [27], antidiabetic [28,29,30], antioxidant [12,16,20,21,31], carbonic anhydrize inhibitory [26,32,33], anticonvulsive [34,35], antifungal [33,36,37] and many other [38,39] activities. Additionally, the benzothiazole scaffold is present in three FDA approved drugs (Figure 1). They are quizartinb, a receptor tyrosine kinase inhibitor, the flutemetamol-diagnostic tool for Alzheimer disease and the drug riluzole for treatment of amyotrophic lateral sclerosis.

On the other hand, the thiazolidinone core attracted the interest of researchers owing to its various degrees of pharmacological and medicinal activities [18,40,41,42,43,44,45,46,47]. Herein, we explore the antimicrobial effects of benzothiazolylthiazolidin-4-one and their in silico mechanistic investigation.

## 2. Results and Discussion

**Figure 2:**
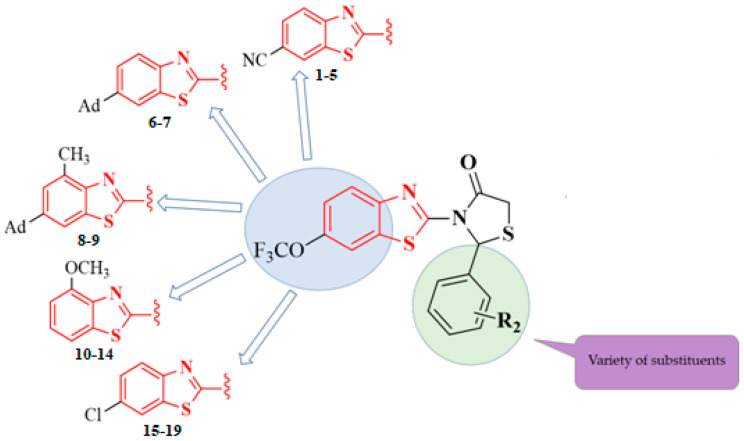
The structural modifications.

Continuing our ongoing research in the field of antimicrobial agents [45,46,47] and based on results of our previous paper [47] we designed new series of compounds modifying the previously synthesized ones. We have replaced 6-trifluoromethoxy substituent of benzothiazole ring first by 6-CN, 6-Ad, 4-Me-6-Ad and after by 4-OCH_3,_ and 6-Cl keeping the same substitution at position 4 of benzene ring mostly for 6-Cl, 4-OMe and in some cases of 6-CN (Figure 2). Thus, we synthesized 2-(2-(substituted phenyl)-4-oxothiazolidin-3-yl)benzo*[d*]thiazole-6-carbonitrile (**1**–**5**), 3-(6-(adamantan-1-yl)benzo[*d*]thiazol-2-yl)-2-(substituted phenyl) thiazolidin-4-one (**6**, **7**), 3-(6-(adamantan-1-yl)-4-methylbenzo*[d*]thiazol-2-yl)-2-(substituted phenyl)thiazolidin-4-one (**8**, **9**), 3-(4-methoxybenzo*[d*]thiazol-2-yl)-2-(4-substituted phenyl)thiazolidin-4-one (**10**–**14**), 3-(6-chlorobenzo*[d*]thiazol-2-yl)-2-(4-substituted phenyl)thiazolidin-4-one (**15**–**19**).

### 2.1. Chemistry

**Scheme 1:**
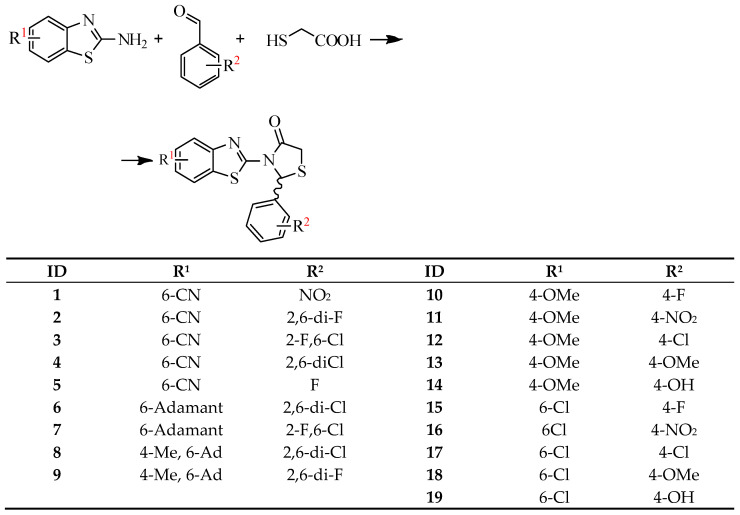
Synthesis of the title compounds.

Compounds were synthesized according to Scheme 1 as published in our previous paper [47].

All products were obtained as racemates and synthesized compounds were characterized by TLC and spectroscopic methods (IR, ^1^H NMR, ^13^C-NMR and MS for some compounds).

In the IR spectra, characteristic strong absorption of the carbonyl vibration in the range of 1700 cm^−1^ and absorption in the 1600 and 1540 cm^−1^ corresponding to the C–C bond of the aromatic ring was observed. The C–H bond of the aromatic ring occurs close to 3000 cm^−1^, while the tertiary amine occurs at 2340–2360 cm^−1^. Finally, the chlorine atoms of phenyl appeared to be poorly absorbed at about 721 and 1100 cm^−1^, respectively**.**

In ^1^H-NMR spectra, signals at 7.00–8.12 ppm, 6.72–7.15 ppm N–CH–S and 3.85–4.13 ppm attributed to aromatic, N–CH–S and –CH_2_ protons, respectively, were observed. It is worth noting that the protons of the 5 position show two characteristic peaks, each one, double split. This is because these two protons are cleaved together as they are neither chemically nor magnetically equivalent. In cases of methoxy-substitution at the benzothiazole or benzene ring, a peak at 3.76–3.95 ppm was observed, while hydroxy-derivatives showed a wide peak at 5.32–5.35 ppm. Finally, the presence of adamantane as a substituent on the benzothiazole ring was confirmed by two peaks at 1.89–1.44 ppm. The first one corresponds to the protons of the three tertiary carbon atoms, while the second peak to the twelve protons of the six tertiary carbon atoms of adamantane.

The ^13^C-NMR peak attributed to the C=O group was observed at 170–171 ppm, while for the C-2 of the benzothiazole ring at 163–165 ppm and for C-2 and C-5 of thiazolidinone moiety at 60–63 ppm and at 31–34 ppm, respectively. The signal of adamantane’s carbon atoms were observed at 41–44, 32–36 and 27–28.5 ppm. Finally, peak attributed to the carbon atom of benzene attached to hydroxyl appeared at 156 ppm (see experimental).

### 2.2. Toxicity Prediction

Taking into account the importance of prediction of toxicity in drug design two software applications Protox and ToxPredict from Open Tox designed according to REACH legislation requirements were utilized in this study [48,49]. The data are presented in Table 1 and Table 2. These software predict probability of carcinogenicity and mutagenicity in various organisms using in silico models and the most accurate estimation of the mean lethal dose (LD_50_) administered to rodents. The reliable estimates are considered to be more than 0.025. All derivatives showed confidence from 0.026 to 0.041 and LD_50_ of 500–1000 mg/kg or higher belonging to group four according to Globally Harmonized System (GHS) of Labeling and Chemicals’ Classification [50] and considered safe for biological experiments. The results of the prediction are presented in Table 1 and Table 2. It is worth mentioning that the prediction accuracy augments as the confidence values increases. Specifically, reliable estimates are regarded to be more than 0.025.

According to Lasar model throughout OpenTox, all the compounds found to be at the category IV with LD_50_ between 500 and 1000 mg/kg and they are safe for use.

### 2.3. Prediction of Activity Spectra of Compounds by Program PASS

PASS prediction of antibacterial activity was performed for the whole set of designed molecules, which were chosen for synthesis and biological testing. Antibacterial activity for all compounds was predicted with the probability to be active Pa values ranging from 0.224 to 0.337 (Table S1). The mechanism of antibacterial activity was predicted as well. The prediction revealed that muramoyltetrapeptide carboxypeptidase inhibition is estimated with Pa between 0.314 and 0.607. The calculated Pa values for all compounds were less than 0.5, indicating their relative novelty compared to the structures of the compounds from the PASS training set [51]. Thus, it can be concluded that the studied compounds have some features different from those of well-known antibacterial agents, which may indicate their innovative potential.

### 2.4. Biological Evaluation

Compounds **1**–**9**, derivatives of 6-CN, 6-Ad, 4-Me-6-Ad benzothiazole based thiazolidinones were evaluated for antibacterial activity, by microdilution method to determine the minimal inhibitory and bactericidal concentrations against the panel of five strains: two Gram positive (*Streptococcus aureus* and *Listeria monocytogenes)* and three Gram negative strains (*Pseudomonas aeruginosa, Escherichia coli* and *Salmonella* *typhimurium*). As reference compounds ampicillin and streptomycin were used. Antibacterial activity of tested compounds is shown in Table 3 with MIC values in the range of 0.10–0.75 mg/mL and MBC at 0.12–1.00 mg/mL. According to the order of activity which can be presented as: **8** > **2** > **4** > **6** > **7** > **1** > **9** > **5** > **3** the highest activity was achieved for compound **8** with MIC at 0.20–0.30 mg/mL and MBC at 0.25–0.50 mg/mL towards non-resistant bacterial strains. The lowest antibacterial potential was observed for compound **3** with MIC values in range of 0.20–0.50 mg/mL and MBC at 0.25–1.0 mg/mL. The most sensitive bacterium appeared to be *E. coli (*ATCC 35210)*,* while *S. typhimirium* was the most resistant one. Four out of nine compounds (**1**, **2**, **4**, **5**) showed very good activity against *E. coli* with MIC/MBC at 0.12/0.25 mg/mL almost equipotent with ampicillin, while compound **2** additionally, as well as compound **6**, demonstrated the same good activity against *S. aureus.* As far as resistant strains are concern the most sensitive to compounds tested appeared to be *P. aeruginosa* and the most resistant MRSA*. P. aeruginosa* was found to be very sensitive to compounds **8**, **4** and **5** with MIC/MBC at 0.06/0.12, 0.20/0.25 and 0.12/0.25 mg/mL, respectively with compound **8** exhibiting almost equipotent activity with streptomycin and higher than ampicillin and **5** being more potent than ampicillin. MRSA was more sensitive to compounds **2**, **4** with MIC/MBC at 0.25/0.50.mg/mL and **3**, **5** with MIC/MBC at 0.30/0.50 mg/mL, whereas *E. coli* demonstrated the same sensitivity to all compounds tested. It should be noticed that streptomycin showed only bacteriostatic activity against MRSA and not bactericidal, while ampicillin was totally inactive against MRSA, in comparison with tested compounds. Interestingly, our compounds manifested good efficiency against ampicillin resistant *P. aeruginosa* and *E. coli* as well as against streptomycin resistant MRSA.

According to the structure–activity relationship studies the presence of 4-CH_3_, 6-adamantyl substituents in benzothiazole ring in combination with 2,6-di-Cl substituents in benzene ring (**8**) seems to be beneficial for antibacterial activity of these group of compounds. Introducing CN group in position 6 of benzothiazole ring and 2-6-di-F substituents in benzene (**2**) decreased slightly the activity, while replacement of 2,6-di-F by 2,4-di-Cl substituents led to less active compound (**4**), which nevertheless is considered as active. On the other hand, introduction of 2-F,6-Cl substituents in benzene ring appeared to be detrimental. The analysis of structure–activity relationships revealed that antibacterial activity of these compounds depends on substituents on benzene ring as well as on benzothiazole one. Thus, in case of 6-CN substituted derivatives the most active is 2,6-di-F, followed by 2-4-di-Cl and the last is 2-F,6-Cl, while for 6-adamantane, 4-CH_3_ and 6-adamantyl substitution in benzothiazole moiety the presence of 2,6-di-Cl in benzene ring is beneficial.

The comparison, of obtained results on antibacterial activity with those of compounds with 6-OCF_3_ substituent in benzothiazole ring revealed that in the case of 4-F substitution in benzene ring the replacement of 6-OCF_3_ by 6-CN improved the activity only against resistant strain of *P. aeruginosa*, while the presence of the 4-NO_2_ group in the benzene ring was beneficial for the resistant strain of *E. coli*. Better improvement was observed in case of 2,6-di-F substitution, namely against *S. aureus,* MRSA*, L. monocytogenes* and resistant strain of *E. coli* up to 2, 2.7 fold, respectively The presence of 2-F,6-Cl substituent almost did not influence the activity, while 2,6-di-Cl substituent improved 2.5 fold the activity against *E. coli*. As for the adamantine moiety occupies the 6-position, introduction of 2,6-di-Cl substituent was beneficial for activity against *S. aureus* improving it two times; while for 4-Me-6-adamantane series, 2,6-di-Cl substituent induced a 2-fold increase in efficiency against resistant strain of *P. aeruginosa.* The same beneficial effect was observed against *S. aureus* with the presence of 2,6-di-F substitution in this series. Thus, it can be concluded that replacement of 6 OCF_3_ substituent by 6-CN improved the activity in most cases by 2–2.7 times.

In an attempt to improve more the antibacterial activity, we decided to introduce in position 4 and 6 of benzothiazole ring methoxy (**10**–**14**) and chloro (**15**–**19**) substituents, respectively.

Compounds **10**–**19** were evaluated for their antibacterial activity against the same bacterial strains. The results are presented in Table 4 and MIC values are in the range of 0.06–0.75 mg/mL and MBC at 0.12–1.00 mg/mL. As already mentioned, all compounds showed antibacterial activity with the following order: **18** > **16** > **19** > **15** > **14** > **17** > **10** > **11** > **13** > **12**. The best activity was achieved for compound **18** with MIC and MBC at 0.10–0.25 mg/mL and 0.12–0.5 mg/mL, respectively, while compound **12** showed the lowest one (MIC/MBC at 0.25–0.50/0.50–1.00 mg/mL) towards non-resistant strains tested. The most sensitive bacterium again was *E. coli* (ATCC 35210), followed by *P. aeruginosa*, while *L. monocytogenes* was the most resistant one. Compounds **15**, **17** and **19** exhibited good activity against Gram negative bacterium *P. aeruginosa* with MIC/MBC at 0.12/0.25 mg/mL, while compound **18** demonstrated very good activity (MIC/MBC at 0.10/0.12 mg/mL) being all of them almost equipotent with streptomycin and twice more potent than ampicillin. Additionally, **18** showed good activity, better than both reference drugs, also against the most sensitive and most resistant bacterial strains (*E. coli* and *L. monocytogenes*) with MIC/MBC at 0.12/0.20 mg/mL. A little bit lower activity against *E. coli* was observed for **10** and **13** with MIC and MBC at 0.12 and 0.25 mg/mL, respectively. Good activity against *S. aureus* and *S. typhimurium* with MIC and MBC at 0.10 mg/mL and 0.12 exhibited compound **16**. It should be mentioned that this compound appeared to be the most potent against *L. monocytogenes* with MIC/MBC at 0.06/0.12 mg/mL, followed by compound **19** being both more active than reference drugs. It was observed that in general compounds **15**–**19** were found to be the most potent among all tested, with compound **18** to be equipotent with streptomycin against almost all bacteria strains tested except *S. aureus* and *S. typhimurium*.

These compounds were also tested against three resistant bacterial strains: MRSA, *P. aeruginosa* and *E. coli.* All of them exhibited activity against MRSA with MIC at 0.10–0.75 mg/mL and MBC in range of 0.12–>1.00 mg/mL. The best activity was shown by compound **16** (MIC/MBC at 0.10/0.12 mg/mL) followed by compound **18** with MIC and MBC at 0.20 mg/mL and 0.25 mg/mL, respectively, while compound **12** was the less potent**.** It should be mentioned that activity of compounds against MRSA was superior to reference drugs. Thus, streptomycin showed only bacteriostatic activity with MIC at 0.10 mg/mL, while ampicillin did not exhibit neither bacteriostatic nor bactericidal activities. As far as activity against resistant *P. aeruginosa* is concerned, compounds appeared to be very potent with MIC at 0.06–0.25 mg/mL and MBC in range of 0.12–0.50 mg/mL. It should be mentioned that even the less potent compounds **10**–**14** appeared to be very potent against this bacterium strain with MIC ranging from 0.12–0.25 and MBC at 0.25–0.50 mg/mL. The most potent was found to be compound **18** (MIC/MBC at 0.06/0.12 mg/mL), followed by compounds **17** and **19** with MIC/MBC at 0.10/0.12 mg/mL. All compounds showed higher potential than ampicillin against *P. aeruginosa* with compound **18** being almost equipotent with streptomycin (MIC/MBC at 0.05/0.10 mg/mL) and 3-fold more active than ampicillin. Regarding resistant *E. coli* it was found that all compounds were more active than ampicillin (MIC at 0.20 mg/mL, without bactericidal activity). The best activity exhibited compounds **16** and **19** with MIC and MBC at 0.12 mg/mL and 0.25 mg/mL, respectively being almost equipotent with streptomycin.

In summary, all compounds were more potent than ampicillin against MRSA, while compounds **16**, **19** and **18** appeared to be equipotent with streptomycin against resistant strains *E. coli* and *P. aeruginosa* respectively.

Compounds with the most promising antibacterial potential were studied for their effect on biofilm formation (Table 5). Despite that none of the tested compounds exhibited activity better than reference drugs in concentration of MIC, compound. **19** demonstrated the highest antibiofilm potency being, in concentration of half MIC better than streptomycin by 1.5 fold.

The structure–activity relationship revealed that the presence of 6-Cl substitution in benzothiazole ring is more beneficial than 4-OCH_3_ one. However, antibacterial activity of these compounds depends not only on substitution at the benzothiazole ring but on the combination of substituents at the benzothiazole moiety and benzene ring as well. Thus, the presence of 6-Cl substituent in combination with 4-OCH_3_ of benzene ring (**18**) appeared to be the most favorable. Replacement of 4-OCH_3_ by 4-NO_2,_ led to a slightly less active compound **16**. The third best compound was found to be **19** with 4-OH substitution in benzene ring, while the less active was compound **12** with 4-OCH_3_ of benzothiazole and 4-Cl substituent on benzene ring. It should be mentioned that the presence of 4-Cl substitution independent of 6-Cl or 4-OCH_3_ on benzothiazole ring was detrimental. The comparison of the activity of compounds **15**–**19** and **10**–**14** revealed that 4-OH substitution was beneficial in case of series with 4-OCH_3_ substitution in benzothiazole ring, while for compounds with 6-Cl at benzothiazole moiety compound **19** was third in order of activity. In general, it was observed the opposite activity of compounds with the same substituents in benzene ring, but different at benzothiazole ring. Thus, in case of 6-Cl benzothiazole derivatives activity can be presented as: 4-OCH_3_ > 4-NO_2_ > 4-OH > 4-F > 4-Cl, whereas in case of 4-OCH_3_ benzothiazole derivatives it is: OH > F > NO_2_ > OCH_3_ > Cl. The only common is that the presence of 4-Cl substituent in benzene ring is detrimental for antibacterial activity in both cases.

The comparison of obtained results on antibacterial activity with those of compounds with 6-OCF_3_ substituent in benzothiazole ring [47] revealed that in case of 4-F substitution on benzene ring the replacement of 6-OCF_3_ by 6-Cl improved twice the activity against *P. aeruginosa* and *P. aeruginosa* resistant, while the replacement by 4-OMe slightly improve only activity against *E. coli* (0.15 and 0.12 mg/mL). Against other species, these replacements did not improve the activity but decreased 3/2 times it in case of MRSA, *L. monocytogenes* and *S typhimurium* against resistant strain of *E. coli* respectively. Among 4-nitro derivatives the presence of 6-Cl substituent in benzothiazole ring appeared to be beneficial compared to 6-OCF_3_ and 4-OCH_3,_ since activity against *S. aureus*, MRSA and resistant strain of *E. coli* increased 2.5 fold, while against *L. monocytogenes* and *S. typhimurium* from 4 to 5 fold. Better results (2–3 times) were obtained in case of the presence of 4-OMe and 4-OH substituents in benzene ring of 6-Cl-benzothiazole derivatives compared to the same substituents at the 4 position of benzene ring of 4-OCF_3_ derivatives.

From all mentioned above it is obvious that modifications performed (replacement of 6-OCF_3_ by 6-Cl in benzothiazole ring), improved the activity against some species from 2 to 5 fold.

### 2.5. Antifungal Activity

Compounds **1**–**9** were tested for their possible antifungal activity (Table 6), which was moderate to low and can be presented in following descending order: **6** > **9** > **1** = **7** > **8** > **2** = **3** = **4** > **5**. Despite, in general these compounds were modestly effective; some of them demonstrated good activity against some fungi species. Thus, compounds **1** and **9** (MIC/MFC of 0.25/0.50 mg/mL) showed activity almost equal to ketoconazole (MIC/MFC of 0.20/0.50 mg/mL) against *Aspergillus versicolor*, while **6** against *Penicillium funiculosum and Penicillium verrucosum var. cyclopium,* at the same time exhibiting 3 fold higher activity than ketoconazole against *Trichoderma viride.* The same activity against this fungal was shown by compound **9**_._

Replacement of 6-CN group by 4-OMe of benzothiazole moiety did not improve much the antifungal activity, while the presence of 6-Cl substituent increased it but still being lower comparing with antibacterial. The order of activity can be presented as follows: **19** > **18** > **16** > **17** > **10** = **11** > **14** > **12** = **13** > **15**. The best activity among compounds tested was achieved for compound **19** with MIC in range of 0.12–0.50 mg/mL and MFC at 0.25–1.00 mg/mL, while the lowest effect was observed for compound **15** (MIC from 0.25 to >1.00 mg/mL and MFC from 0.5 to >1.00 mg/mL). Compounds **18** and **19** exhibited the highest potency, twice better/equipotent than that of ketoconazole against *A. versicolor* and *P.v.c*., respectively with MIC at 0.12 mg.ml and MFC at 0.25 mg/mL, while compound **16** showed the same good activity against *Aspergillus fumigatus.* Furthermore, all compounds demonstrated good activity against *T. viride* being superior to ketoconazole with MIC/MFC at 1.0/1.5 mg/mL. Compounds **19**, **10** and **14** were almost equipotent with ketoconazole against *A. versicolor,* while **19,** additionally**,** against *P. funiculosum*. The most sensitive fungal appeared to be *T. viride*, while *Aspergillus niger* was the most resistant one.

The structure–activity relationships study showed that the presence of hydroxyl group at position 4 of benzene ring (**19**) is beneficial for antifungal activity. Replacement of hydroxyl by methoxy group led to a little less potent compound **18**. Introduction of nitro or fluoro group at 4-position of benzene ring derivatives (**16**) or (**15**) had negative influence on antifungal activity, the latter being the less active compound. In case of compounds with methoxy group at position 4 of benzothiazole moiety, the presence of fluoro- and nitro substituents at position 4 of benzene ring demonstrated the same influence on antifungal activity, as compounds **15**–**19.** In group of 6-CN-benzothiazole based thiazolidinones, the presence of 4-F was detrimental, while nitro substituent showed the same behavior like in two groups mentioned above. The comparison between the results of these two series of compounds **15**–**19** with 6-Cl substitution in benzothiazole moiety and **10**–**14** revealed that antifungal activity of compounds depends not only on the nature of the substituent of benzene ring but also on the nature and position of substituent of benzothiazole ring.

### 2.6. In Silico Predictive Studies (Molecular Properties and Drug-Likeness)

Drug likeness is examined as an important part that provides the base for the molecules to be a powerful drug candidate. There are several rules, such. Lipinski [52], Ghose [53], Veber [54], Egan [55], and Muegge [56] can be used to measure drug-likeness of the candidate compounds according to some acute criterion. These criteria are a molecular weight, Log *P*, number of hydrogen bond acceptors and donors.

Molecular properties viz., bioavailability and membrane permeability are correlated with simple molecular descriptors such as partition coefficient log *P*, H-bond donors and acceptors in a molecule [56]. Lipinski’s rule [52] of 5 is employed to disclose “drugability” of molecules. Thus, only for two compounds **6** and **8** molecular weights was higher than 500. Violations to the above-revealed rules together with drug-likeness and oral bioavailability scores are represented in Table 7. Most of the compounds violated any rule and their bioavailability score was around 0.55. The absorption magnitude is given as an absorption percentage. Following the law %ABS = 109 − 0.345 PSA, the absorption percent was computed [57]. Polar surface area (PSA) was defined as the fragment-based increments described by Ertl and coworkers [58,59]. The existence of more than 10 hydrogen-bond acceptors, 5 hydrogen-bond donors, demonstrates poor absorption or permeation. All derivatives contain <10 hydrogen bond acceptors and <5 hydrogen bond donors (Table 7).

As depicted in figures of Table 8, curves with green color indicates non-drug-like behavior and blue color are considered as drug-like. Compounds with zero or negative value cannot be considered as drug-like. The drug-likeness score was found to be from −0.42 to 0.56 for the compounds under investigation. However, compounds **6** and **8** have two violations from Lipinski rule and cannot be treated as drug candidate; even they showed good antibacterial activity.

### 2.7. Docking Studies

#### Docking Studies to Antibacterial Targets

According to PASS prediction, our compounds found to be possible inhibitors of LD-carboxypeptidase (LdcA). As such, we included this enzyme to docking studies. LD-Carboxypeptidases acts by cleaving amide bonds between L- and D-amino acids in bacterial peptidoglycan. More specific, cleaving the link between *meso*-diaminopimelic acid and d-alanine and consequently reduce tetrapeptides to the corresponding tripeptides, which can then be reconverted into peptidoglycan building blocks by the attachment of preformed D-Ala-D-Ala dipeptides. Therefore, LD-carboxypeptidases are thought to play a critical role in peptidoglycan recycling [60]. Crystallographic studies revealed that LD-carboxypeptidase is a serine protease with that Ser^115^, His^285^, and Glu^217^ forming a functional catalytic triad [61].

The docking studies showed that the free energy of binding to *E. coli* DNA Gyrase, Thymidylate kinase, *E. coli* Primase and *E. coli* MurB were higher than that to LdcA, therefore it may be considered that inhibition of LdcA enzyme is probably the possible mechanism of action of the compounds (Table 9).

**Figure 3:**
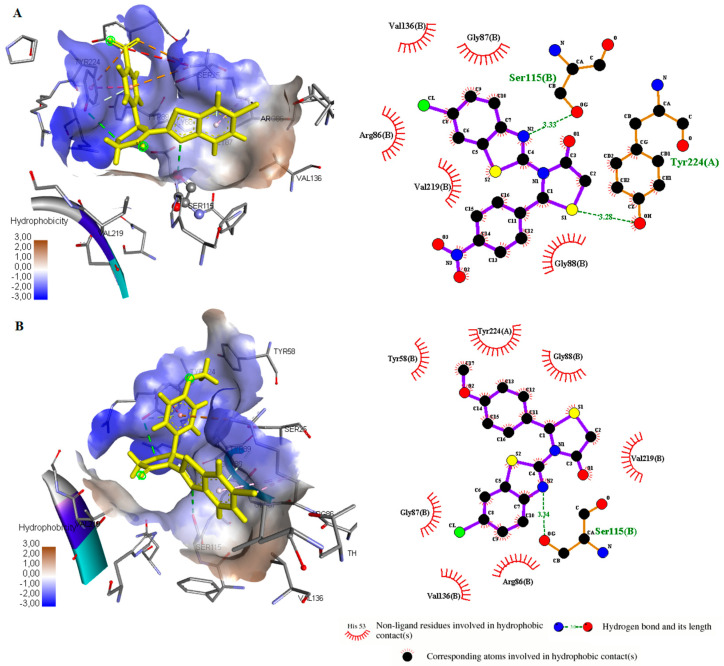
Docked conformations of the two most active compounds: **A**-**16** and **B-18** in LdcA enzyme and 2D diagrams of ligand–enzyme interactions. Green dotted lines represent hydrogen bond.

Docking studies revealed that the most active compound **18** binds to LdcA enzyme forming a favorable hydrogen bond interaction between the nitrogen atom of benzothiazole ring and the hydrogen of the side chain of Ser116 (distance 3.24 Å). The benzothiazole moiety interacts hydrophobically with the residues Val36, Arg86 and Gly87, while the thiazolidinone ring with the residues Gly88 and Val219 (Figure 3B). Furthermore, the benzene moiety is placed in a cavity that consists of the residues Tyr58, Gly88 and Tyr224, interacting hydrophobically. These interactions further stabilize the complex compound-enzyme contributing to inhibitory activity of the compound **18**. Moreover, the hydrogen bond formation with the residue Ser115 is crucial for the inhibitory activity of this compound, as it is among the amino acids of the catalytic triad of the enzyme. Hydrogen bond interactions with the residues of the catalytic triad of the enzyme were also observed for compounds **16** (Figure 3B), **19**, **2**, **4**, **6** and **8**, explaining their higher inhibitory activity.

Docking studies to antifungal targets showed that only compounds **18** and **19** had a significant good estimated free energy of binding to CYP51ca enzyme with values −6.78 and −6.94 kcal/mol respectively. The rest of the compounds had values ranging from −2.10 to −6.21 kcal/mol.

## 3. Materials and Methods

The MEL-TEMP II device (LAB Devices, Holliston, MA, USA) was used to determine the melting points and are uncorrected. Infrared (IR) spectra were recorded in Nujol on the Perkin Elmer Spectrum BX dual-beam spectrometer. ^1^H NMR nuclear magnetic resonance spectra in DMSO-d6 or CDCl3 were obtained with an Agilent spectrometer at 500 MHz. Chemical shift values are given in parts per million (ppm/s), while tetramethylsilane (TMS, δTMS = 0) was used as the internal standard. The ESI-MS (Micromass ZMD Waters) spectrometer was used to obtain the mass spectra (MS). The progress of the reactions was checked by thin layer chromatography using F254 silica gel chromatography plates (Merck, Darmstadt, Germany). All reagents and solvents were purchased from Aldrich Chemie (Steinheim, Germany) and were of high analytical purity.

### 3.1. Chemistry

Method A. The appropriate (hetero) aromatic amine (1.0 mmol) and the appropriately substituted benzaldehyde (1.2 mmol) were refluxed in dry toluene followed by the addition of thioglycolic acid (2.0 mmol). Heating is continued for 4–39 h until the (hetero) aromatic amine complete reacted. At the end of the reaction the solvent was removed in vacuo and the residue was taken up in ethyl acetate. This is followed by successive washes of the organic layer with 5% aqueous citric acid, water and 5% aqueous sodium bicarbonate. The solvent was removed in vacuo and the organic layer was dried over sodium sulfate. A solid residue was obtained and washed with 95% ethanol. The final product was allowed to dry and recrystallized from 95% ethanol if necessary.

Method B. The reactions using microwave radiation were performed with the CEM-Discover Monomode instrument, with a frequency of 2.45 GHz and continuous irradiation with a maximum power of 100 W. The appropriate (hetero) aromatic amine (1 mmol) together with the appropriately substituted benzaldehyde (1.3 mmol) and thioglycolic acid (5 mmol) were placed in a special tube with a capacity of 10 mL. Add 2–3 mL of absolute ethanol, covered the tube with a special Teflon stopper and placed it in the instrument (CEM). The mixture was irradiated for 20–30 min at 80–100 °C using a maximum pressure of 250 psi. The reaction stirred continuously and after completion, the tube was cooled to ambient temperature. The solid product was filtered under reduced pressure, washed with methanol and allowed to dry.

Synthesis of 2-(2-(4-nitrophenyl)-4-oxothiazolidin-3-yl)benzo[*d*]thiazole-6-carbonitrile (**1**).

Method A: The reaction time was 13 h. Yield: 54.4% Method B (MW irradiation): The reaction was carried out at a temperature of 100 °C and the time required was 30 min. Yield: 82.5% M. p.: 166–167 °C. Rf: 0.62 (petroleum ether/ethyl acetate: 8/2). IR: (cm^−1^, Nujol): 2364 (=Ν-), 2218 (-CN), 1706 (C=O). ^1^H-NMR (500 MHz, CHCl_3_-d): δ ppm 3.91 (d, *J* = 16.63 Hz, 1H, CH_2_), 4.11 (d, *J* = 16.63 Hz, 1H, CH_2_), 6.49 (s, 1H, N-CH-S), 7.48 (d, *J* = 7.83 Hz, 2H, Ar-C17, C21), 7.55–7.57 (m, 1H, Ar-C5), 7.77 (d, *J* = 7.86 Hz, 1H, Ar-C4), 8.20 (d, *J* = 7.81 Hz, 2H, Ar-C18, C20), 8.40 (d, *J* = 7.83 Hz, 1H, Ar-C7). ^13^C-NMR (500 MHz, CHCl_3_-d): δ ppm 32.77 (1C, CH_2_), 62.57 (1C, N-CH-S), 103.88, 115.42 (2C, Ar), 118.80 (1C, -CN), 122.52, 124.30(2C), 126.41(2C), 133.25, 142.24, 147.80, 153.69, 157.25 (10C, Ar), 163.70 (1C, N=C-–N), 170.28 (1C, C=O).

Synthesis of 2-(2-(2,6-difluorophenyl)-4-oxothiazolidin-3-yl)benzo[***d***]thiazole-6-carbonitrile (**2**).

Reaction time: 10 h. Yield: 57%. M p.183–184°C. Rf: 0.71 (petroleum ether/ethyl acetate: 8/2). IR: (cm^−1^, Nujol): 2361 (=Ν-), 2218 (-CN), 1711 (C=O), 1591 (C=C arom), 1001 (C-F). ^1^H-NMR (500 MHz, CHCl_3_-d): δ ppm 3.95 (d, *J* = 16.14 Hz, 1H, CH_2_) 4.11 (d, *J* = 16.14 Hz, 1H, CH_2_) 6.80 (s, 1H, N-CH-S) 7.31–7.40 (m, 2H, Ar-C18, C20) 7.42–7.49 (m, 1H, Ar-C19Ar-C18, C20) 7.53 (d, *J* = 8.31 Hz, 1H, Ar-C5) 7.92(d, *J* = 8.31 Hz, 1H, Ar-C4) 8.02 (d, *J* = 1.96 Hz, 1H, Ar-C7). ^13^C-NMR (500 MHz*,* CHCl_3_-d): δ ppm 33.20 (1C, CH_2_), 60.11 (1C, N-CH-S), 104.43, 111.07(2C), 112.85, 117.65 (5C, Ar), 118.25 (1C, -CN), 126.72, 128.71, 129.47, 130.48, 145.72, 158.02 (7C, Ar), 163.44 (2C, C-F), 163.59 (1C, N=C-N), 171.01 (1C, C=O).

Synthesis of 2-(2-(2-chloro-6-fluorophenyl)-4-oxothiazolidin-3-yl)benzo[*d*]thiazole-6-carbonitrile (**3**).

Reaction time: 16 h (A), 30 min (B) respectively. Yield: 41.5%(A), 88.5% (B), respectively. M p.176–177 °C. Rf: 0.76 (petroleum ether/ethyl acetate: 8/2). IR: (cm^−1^, Nujol): 2361 (=Ν-), 2218 (-CN), 1709 (C=O), 1602 (C=C arom), 1191 (C-F), 721 (C-Cl). ^1^H-NMR (500 MHz, CHCl_3_-d): δ ppm 4.04 (d, *J* = 16.63 Hz, 1H, CH_2_), 4.28 (d, *J* = 16.63 Hz, 1H, CH_2_), 6.84 (s, 1H, N-CH-S), 7.09 (t, *J* = 8.43 Hz, 1H, Ar-C19), 7.59–7.65 (m, 2H, Ar-C18, C20), 7.72 (d, *J* = 7.32 Hz, 1H, Ar-C5), 7.93 (d, *J* = 7.32 Hz, 1H, Ar-C4), 8.37 (d, *J* = 8.32 Hz, 1H, Ar-C7). ^13^C-NMR (500 MHz, CHCl_3_-d): δ ppm 33.72 (1C, CH_2_), 60.28 (1C, N-CH-S), 104.49, 113.67, 117.57 (2C, Ar), 118.19 (1C, -CN), 125.31, 126.87, 128.79, 129.85, 130.81, 131.47, 135.29, 157.02 161.18 (9C, Ar), 163.74 (1C, N=C-N), 171.23 (1C, C=O).

Synthesis of 2-(2-(2,6-dichlorophenyl)-4-oxothiazolidin-3-yl)benzo[*d*]thiazole-6-carbonitrile (**4**).

Reaction time: 18 h (A), 30 min (B) respectively. Yield: 29.1%(A), 83.4% (B), respectively. M p.198–200 °C. Rf: 0.63 (petroleum ether/ethyl acetate: 8/2). IR: (cm^−1^, Nujol): 1706 (C=O), 1572, 1230, 1213. ^1^H-NMR (500 MHz, CHCl_3_-d): 3.76 (s, 3H, O-CH_3_), 3.91 (d, *J* = 16.38 Hz, 1H, CH_2_), 4.10 (d, *J* = 16.63 Hz, 1H, CH_2_), 6.73 (s, 1H, N-CH-S), 6.84 (d, *J* = 8.80 Hz, 2H, Ar-C18, C20), 7.12 (t, *J* = 8.56 Hz, 1H, Ar-C5), 7.23–7.35 (m, 1H, Ar-C4), 7.46–7.55 (m, 2H, Ar-C17, C21), 7.58–7.79 (m, 1H, Ar-C7). ^13^C-NMR (500 MHz, CHCl_3_-d): δ ppm 33.72 (1C, CH_2_), 58.01, 63.08, 109.98, 113.89, 114.13, 117.94, 129.49, 131.98, 132.35, 137.38, 146.73, 156.1, 158.01, 163.51, 171.03 (1C, C=O).

Synthesis of 2-(2-(4-fluorophenyl)-4-oxothiazolidin-3-yl)benzo*[d]*thiazole-6-carbonitrile (**5**).

Reaction time: 25 h (A), 30 min (B) respectively. Yield: 52.4%(A), 87.1% (B), respectively. M p.185–186 °C. Rf: 0.68 (petroleum ether/ethyl acetate: 8/2). IR: (cm^−1^, Nujol): 2364 (=Ν-), 2218 (-CN), 1709 (C=O). ^1^H-NMR (500 MHz***,*** CHCl_3_-d): 3.94 (d, *J* = 16.14 Hz, 1H, CH_2_), 4.10 (d, *J* = 16.14 Hz, 1H, CH_2_), 6.82 (s, 1H, N-CH-S), 7.12–7.22 (m, 4H, Ar-C17, C18, C20, C21), 7.81 (d, *J* = 8.31 Hz, 1H, Ar-C5), 7.92 (d, *J* = 8.31 Hz, 1H, Ar-C4) 8.16 (d, *J* = 1.96 Hz, 1H, Ar-C7). ^13^C-NMR (500 MHz, CHCl_3_-d): δ ppm 33.21 (1C, CH_2_), 64.14, 104.41, 115.45, 117.65, 118.23, 126.72, 129.47, 130.55, 131.09, 134.18, 158.15, 161.25, 164.69, 170.92 (1C, C=O).

Synthesis of 3-(6-(adamantan-1-yl)benzo[*d*]thiazol-2-yl)-2-(2,6-dichlorophenyl)thiazolidin-4-one (**6**).

Reaction time: 21 h (A), 30 min (B) respectively. Yield: 88.3% (A), 89.7% (B), respectively. M p.184–185 °C. Rf: 0.45 (petroleum ether/ethyl acetate: 8/2). IR: (cm^−1^, Nujol): 2974, 2879, 2840, (C-H adamant), 2347 (=Ν-), 1700 (C=O), 1532 (C=C arom), 721 (C-Cl). ^1^H-NMR (500 MHz, CHCl_3_-d): δ ppm 1.75–1.82 (m, 3H, Ad), 1.85–1.93 (m, 3H, Ad), 1.95–2.01 (m, 3H, Ad), 3.91 (br d, *J* = 16.14 Hz, 1H, CH_2_), 4.13 (d, *J* = 16.14 Hz, 1H, CH_2_), 6.80 (s, 1H, N-CH-S), 7.45–7.48 (m, 3H, Ar-C18, C19, C20), 7.93 (d, *J* = 8.31 Hz, 1H, Ar-C4), 8.04 (d, *J* = 1.96 Hz, 1H, Ar-C7). ^13^C-NMR (500 MHz, CHCl_3_-d): δ ppm 28.93 (3C, Ad), 32.75 (1C, CH_2_), 36.46(1C, Ad), 36.80(3C, Ad), 43.42(3C, Ad), 62.15 (1C, N-CH-S), 119.58, 121.05, 124.11, 128.39(2C), 129.55, 130.98, 135.42(2C), 139.11, 142.03, 150.54 (12C, Ar), 164.13 (1C, N=C-N), 171.10 (1C, C=O).

Synthesis of 3-(6-(adamantan-1-yl)benzo[*d*]thiazol-2-yl)-2-(2-chloro-6-fluorophenyl) thiazolidin-4-one (**7**).

Reaction time: 15 h (A). Yield: 73.4%(A). M p.183–184 °C. Rf: 0.59 (petroleum ether/ethyl acetate: 8/2). IR: (cm^−1^, Nujol): 2843 (C-H Ad), 2358 (=Ν-), 1653 (C=O), 1534 (C=C arom), 1099 (C-F), 724 (C-Cl). ^1^H-NMR (500 MHz, CHCl_3_-d): δ ppm 1.75–1.82 (m, 3H, Ad), 1.93 (d, *J* = 2.45 Hz, 3H, Ad) 2.11 (br s, 3H, Ad) 3.91 (br d, *J* = 16.14 Hz, 1H, CH_2_), 4.27 (d, *J* = 16.14 Hz, 1H, CH_2_), 6.80–6.90 (m, 2H, Ar-C18, C20), 7.10 (s, 1H, N-CH-S), 7.15–7.24 (m, 1H, Ar-C19) 7.42 (dd, *J* = 8.80, 1.96 Hz, 1H, Ar-C5), 7.65 (d, *J* = 8.31 Hz, 1H, Ar-C4), 7.74 (d, *J* = 1.96 Hz, 1H, Ar-C7). ^13^C-NMR (500 MHz***,*** CHCl_3_-d): δ ppm 28.92 (2C, Ad), 29.68 (1C, Ad), 33.82 (1C, CH_2_), 36.45(1C, Ad), 36.69(3C, Ad), 43.41(3C, Ad), 53.99 (1C, N-CH-S), 111.71, 111.88, 111,90, 117.13, 121.38, 123.51, 128.92(2C), 129.97, 132.14, 146.10, 148.11 (12C, Ar), 155.37 (1C, N=C-N), 170.65 (1C, C=O).

Synthesis of 3-(6-(adamantan-1-yl)-4-methylbenzo[*d*]thiazol-2-yl)-2-(2,6-dichlorophenyl)thiazolidin-4-one (**8**).

Reaction time: 17 h (A). Yield: 81.8% (A). M p.191–192 °C. Rf: 0.43 (petroleum ether/ethyl acetate: 8/2). IR: (cm^−1^, Nujol): 2856 (C-H Ad), 2364 (=Ν-), 1700 (C=O), 1381 (-CH_3_), 721 (C-Cl). ^1^H-NMR (500 MHz, CHCl_3_-d): δ ppm 1.73–1.82 (m, 3H, Ad), 1.85–1.93 (m, 3H, Ad), 1.97–2.02 (m, 3H, Ad), 2.55 (s, 3H, -CH_3_), 3.95 (d, *J* = 16.14 Hz, 1H, CH_2_), 4.12 (d, *J* = 16.14 Hz, 1H, CH_2_), 6.81 (s, 1H, N-CH-S), 7.24 (s, 1H, Ar-C5), 7.40–7.53 (m, 3H, Ar-C18, C19, C20), 7.95 (d, *J* = 1.96 Hz, 1H, Ar-C7). ^13^C-NMR (500 MHz, CHCl_3_-d): δ ppm 16.75(1C, -CH_3_) 28.92 (3C, Ad), 32.74 (1C, CH_2_), 36.44(1C, Ad), 36.80(3C, Ad), 43.42(3C, Ad), 62.47 (1C, N-CH-S), 115.53, 124.17, 124.98, 128.16(2C), 129.17, 131.01, 135.44(2C), 139.12, 142.05, 145.00 (12C, Ar), 164.13 (1C, N=C-N), 171.11 (1C, C=O).

Synthesis of 3-(6-(adamantan-1-yl)-4-methylbenzo*[d*]thiazol-2-yl)-2-(2,6-difluorophenyl)thiazolidin-4-one (**9**).

Reaction time: 22 h (A), 30 min (B) respectively. Yield: 87.3% (A), 89.6% (B), respectively. M p.188–189 °C. Rf: 0.59 (petroleum ether/ethyl acetate: 8/2). IR: (cm^−1^, Nujol): 2842 (C-H Ad), 2352 (=Ν-), 1709 (C=O), 1535 (C=C arom), 1383 (-CH_3_), 1099 (C-F). ^1^H-NMR (500 MHz, CHCl_3_-d): δ ppm 1.73–1.84 (m, 3H, Ad), 1.86–1.93 (m, 3H, Ad), 1.95–2.03 (m, 3H, Ad), 2.54 (s, 3H, -CH_3_), 3.86 (d, *J* = 16.14 Hz, 1H, CH_2_), 4.14 (d, *J* = 16.14 Hz, 1H, CH_2_), 6.85 (s, 1H, N-CH-S), 7.25–7.39 (m, 3H, Ar-C5, C18, C20), 7.59 (br t, *J* = 8.80, 1H, Ar-C19), 7.95 (d, *J* = 1.96 Hz, 1H, Ar-C7). ^13^C-NMR (500 MHz, CHCl_3_-d): δ ppm 16.75(1C, -CH_3_) 28.91 (3C, Ad), 32.74 (1C, CH_2_), 36.41(1C, Ad), 36.92(3C, Ad), 43.41(3C, Ad), 58.86 (1C, N-CH-S), 111.51(2C), 112.74, 115.91, 124.12, 124.76, 128.49(2C), 131.01, 142.33 (10C, Ar), 162.85(2C, C-F), 164.11 (1C, N=C-N), 171.08 (1C, C=O). (MS): (m/z) 497 (M^+^, 12%), 482 (7%), 463 (5%), 423 (78%), 244 (100%), 198 (10%), 171 (11%), 152 (18%).

Synthesis of 2-(4-fluorophenyl) -3-(4-methoxybenzo [*d*] thiazol-2-yl) thiazolidin-4-one (**10**).

Reaction time: 6h (A). Yield:51.4%. M p. 251–252 °C.: Rf: 0.73 (petroleum ether/ethyl acetate: 8/2). IR: (cm^−1^, Nujol): 2364 (=Ν-), 1709 (C=O), 1587 (C=C arom), 1272 (-OCH_3_), 1113 (C-F, benz). ^1^H-NMR (500 MHz, DMSO-d_6_): δ ppm 3.68–3.86 (m, 3H, O-CH_3_), 3.91–4.09 (m, 1H, CH_2_), 4.25 (br d, *J* = 9.78 Hz, 1H, CH_2_), 6.88 (s, 1H, N-CH-S), 7.06 (br d, *J* = 10.27 Hz, 1H, Ar-C5), 7.26 (br dd, *J* = 10.03, 8.07 Hz, 1H, Ar-C6), 7.55 (br d, *J* = 10.27 Hz, 1H, Ar-C7), 7.61–7.76 (m, 2H, Ar-C17, C21), 8.08–8.26 (m, 2H, Ar-C18, C20). ^13^C-NMR (500 MHz, DMSO-d_6_): δ ppm 32.86 (1C, CH_2_), 55.82 (1C, O-CH_3_), 63.18 (1C, N-CH-S), 108.26, 114.11, 115.43(2C), 121.18, 130.33(2C), 131.99, 135.59, 142.31, 150.23, 161.51 (12C, Ar), 163.91 (1C, N=C-N), 171.08 (1C, C=O). (MS): (m/z) 361 (M^+^, 100%), 343 (42%), 321 (36%), 290 (72%), 288 (31%), 279 (39%), 225 (24%).

Synthesis of 3-(4-methoxybenzo*[d*]thiazol-2-yl)-2-(4-nitrophenyl)thiazolidin-4-one (**11**).

Reaction time: 10 h (A). Yield: 63.5%.M p: 298–299 °C. R_f_: 0.48 (petroleum ether/ethyl acetate: 8/2). IR: (cm^−1^, Nujol): 2364 (=Ν-), 1709 (C=O), 1585 (C=C arom), 1272 (-OCH_3_). ^1^H-NMR (500 MHz, DMSO-d_6_): δ ppm 3.77 (s, 3H, O-CH_3_), 4.03 (d, *J* = 17.12 Hz, 1H, CH_2_), 4.25 (d, *J* = 17.12 Hz, 1H, CH_2_), 6.93 (br d, *J* = 10.27 Hz, 1H, Ar-C5), 7.04 (s, 1H, N-CH-S), 7.26 (s, 1H, Ar-C6) 7.56 (d, *J* = 7.83 Hz, 1H, Ar-C17) 7.67 (d, *J* = 8.80 Hz, 2H, Ar-C7, C21) 8.17 (d, *J* = 8.32 Hz, 2H, Ar-C18, C20). ^13^C-NMR (500 MHz, DMSO-d_6_): δ ppm 32.85 (1C, CH_2_), 55.86 (1C, O-CH_3_), 63.21 (1C, N-CH-S), 108.25, 114.12, 121.18, 123.84(2C), 129.66(2C), 131.97, 142.31, 145.26, 146.32, 150.21(12C, Ar), 163.95 (1C, N=C-N), 171.02 (1C, C=O). (MS*)*: (m/z) 388 (M^+^, 100%), 288 (73%), 272 (3%), 225 (42%), 184 (76%), 136 (42%), 115 (31%), 73 (28%).

Synthesis of 2-(4-chlorophenyl)-3-(4-methoxybenzo[*d*]thiazol-2-yl)thiazolidin-4-one (**12**).

Reaction time 8 h (A) Yield: 43.2%. (B) reaction time 30 min. Yield: 89.4%. M p: 218–219 °C. R_f_: 0.53 (petroleum ether/ethyl acetate:8/2). IR: (cm^−1^, Nujol): 2364 (=Ν-), 1706 (C=O), 1594 (C=C arom), 1275 (-OCH_3_), 721 (C-Cl benz). ^1^H-NMR (500 MHz, DMSO-d_6_): δ ppm 3.80 (s, 3H, O-CH_3_), 3.99 (br d, *J* = 17.12 Hz, 1H, CH_2_), 4.23 (br d, *J* = 16.63 Hz, 1H, CH_2_), 6.93 (s, 1H, N-CH-S), 6.91–6.97 (m, 1H, Ar-C5), 7.26 (br t, *J* = 8.07 Hz, 1H, Ar-C4), 7.39 (br d, *J* = 9.29 Hz, 4H, Ar-C17, C18, C20, C21), 7.54 (br d, *J* = 7.83 Hz, 1H, Ar-C7). ^13^C-NMR (500 MHz, DMSO-d_6_): δ ppm 32.82 (1C, CH_2_), 55.88 (1C, O-CH_3_), 63.27 (1C, N-CH-S), 108.25, 114.11, 121.32, 128.73(2C), 130.12(2C), 131.93, 132.75, 137.36, 142.32, 150.22(12C, Ar), 164.03 (1C, N=C-N), 171.00 (1C, C=O).

Synthesis of 3-(4-methoxybenzo[*d*]thiazol-2-yl)-2-(4-methoxyphenyl)thiazolidin-4-one (**13**).

Reaction time 15 h. Yield: 52.1% (A). Reaction time: 30 min. Yield***:*** 78.5%. (B) Mp: 196–197 °C. R_f_: 0.63 petroleum ether/ethyl acetate (8/2). IR: (cm^−1^, Nujol): 2364 (=Ν-), 1703 (C=O), 1569 (C=C arom), 1269 (-OCH_3_). ^1^H-NMR (500 MHz, CHCl_3_-d): δ ppm 3.76 (s, 3H, O-CH_3benz_), 3.94 (d, *J* = 16.63 Hz, 1H, CH_2_), 3.95 (s, 3H, O-CH_3_), 4.13 (d, *J* = 16.63 Hz, 1H, CH_2_), 6.81–6.85 (m, 2H, Ar-C18, C20), 7.93 (s, 1H, N-CH-S), 7.25 (br d, *J* = 9.29 Hz, 3H, Ar-C5, C6, C7), 7.39 (br d, *J* = 7.83 Hz, 2H, Ar-C17, C21). ^13^C-NMR (500 MHz, CHCl_3_-d): δ ppm 32.86 (1C, CH_2_), 55.83 (1C, O-CH_3_), 63.35 (1C, N-CH-S), 108.25, 114.11, 114.76(2C), 121.82, 129.75(2C), 131.55, 131.96, 142.34, 150.23, 160.82(12C, Ar), 164.06 (1C, N=C-N), 171.11 (1C, C=O). (MS): (m/z) 373 (M^+^, 76%), 272 (33%), 243 (56%), 244 (100%), 242 (8%), 165 (5%), 164 (7%), 135 (11%).

Synthesis of 2-(4-hydroxyphenyl)-3-(4-methoxybenzo[*d*]thiazol-2-yl)thiazolidin-4-one (**14**).

Reaction time: 26 h. Yield: 29.0% (A), reaction time: 30 min. Yield: 72.8%. (B). M p: 261–262 °C. R_f_: 0.60 petroleum ether/ethyl acetate (8/2). IR: (cm^−1^, Nujol): 3193 (-OH), 1703 (C=O), 1572 (C=C arom), 1271 (-OCH_3_). ^1^H-NMR (500 MHz, DMSO-d_6_): δ ppm 3.77 (s, 3H, O-CH_3_), 4.03 (d, *J* = 17.12 Hz, 1H, CH_2_), 4.20 (d, *J* = 17.12 Hz, 1H, CH_2_), 5.32 (br s, 1H, -OH), 6.93 (d, *J* = 7.83 Hz, 2H, Ar-C18, C20), 7.05 (s, 1H, N-CH-S), 7.26 (br t, *J* = 8.56 Hz, 1H, Ar-C5), 7.56 (d, *J* = 7.83 Hz, 1H, Ar-C6), 7.67 (d, *J* = 8.80 Hz, 1H, Ar-C7), 8.17 (d, *J* = 8.32 Hz, 2H, Ar-C17, C21). ^13^C-NMR (500 MHz, CHCl_3_-d): δ ppm 32.88 (1C, CH_2_), 55.82 (1C, O-CH_3_), 63.33 (1C, N-CH-S), 108.21, 114.13, 115.81(2C), 121.73, 130.13(2C), 131.52, 131.96, 142.31, 150.22, 156.95(12C, Ar), 164.13 (1C, N=C-N), 171.04 (1C, C=O).

Synthesis of 3-(6-chlorobenzo*[d*]thiazol-2-yl)-2-(4-fluorophenyl)thiazolidin-4-one (**15**).

Reaction time: 8 h (A). Yield: 51.3%. M p.: 196–197 °C. Rf: 0.76(petroleum ether/ethyl acetate: 8/2). IR: (cm^−1^, Nujol): 2364 (=Ν-), 1681 (C=O), 1571 (C=C arom), 1101 (C-F Bz). ^1^H-NMR (500 MHz, CHCl_3_-d): δ ppm 3.85 (d, *J* = 16.38 Hz, 1H, CH_2_), 4.11 (d, *J* = 16.63 Hz, 1H, CH_2_), 6.75 (s, 1H, N-CH-S), 7.00–7.03 (m, 2H, Ar-C18, C20), 7.30–7.34 (m, 3H, Ar-C5, C17, C21), 7.60 (d, *J* = 8.02 Hz, 1H, Ar-C4), 7.76 (s, 1H, Ar-C7). ***^13^C-***NMR (500 MHz, CHCl_3_-d): δ ppm 32.79 (1C, CH_2_), 63.09 (1C, N-CH-S), 115.77, 115.94, 120.83, 122.70, 126.89, 127.49, 127.55, 133.32, 136.12, 146.77, 156.13 (12C, Ar), 163.52 (1C, N=C-N), 170.87 (1C, C=O). (MS): (m/z) 364 (M^+^, 100%), 343 (10%), 299 (10%), 290 (100%), 279 (9%), 184 (11%), 115 (80%), 88 (9%), 73 (94%).

Synthesis of 3-(6-chlorobenzo[*d*]thiazol-2-yl)-2-(4-nitrophenyl)thiazolidin-4-one (**16**).

Reaction time: 12 h (A), 30 min, 100 °C (B), respectively. Yield: 52.7% (A), 88.3% (B) respectively. M p.: 208–209 °C. Rf: 0.62(petroleum ether/ethyl acetate: 8/2). IR: (cm^−1^, Nujol): 2364 (=Ν-), 1704 (C=O), 1591 (C=C arom). ^1^H-NMR (500 MHz, CHCl_3_-d): δ ppm 3.90 (d, *J* = 16.63 Hz, 1H, CH_2_), 4.12 (d, *J* = 16.63 Hz, 1H, CH_2_), 6.78 (s, 1H, N-CH-S), 7.21–7.30(d, *J* = 7.34 Hz, 2H, Ar-C17, C21), 7.53 (br d, *J* = 8.80 Hz, 1H, Ar-C5), 7.80 (br d, *J* = 8.80 Hz, 1H, Ar-C4), 8.20 (d, *J* = 8.31 Hz, 3H, Ar-C7, C18, C20). ^13^C-NMR (500 MHz, CHCl_3_-d): δ ppm 32.86 (1C, CH_2_), 62.55 (1C, N-CH-S), 121.09, 122.76, 124.42 (2C), 126.37, 127.20(2C), 130.93, 133.31, 145.21 146.85, 151.28 (12C, Ar), 163.55 (1C, N=C-N), 170.48 (1C, C=O).

Synthesis of 3-(6-chlorobenzo[*d*]thiazol-2-yl)-2-(4-chlorophenyl)thiazolidin-4-one (**17**).

Reaction time: 28 h (A), 30 min, 100 °C (B), respectively. Yield: 44.2% (A), 72.1% (B) respectively. M p.: 188–189 °C. Rf: 0.71 (petroleum ether/ethyl acetate: 8/2). IR: (cm^−1^, Nujol): 2358(=Ν-), 1709 (C=O), 1591 (C=C arom), 720 (C-Cl bz). ^1^H-NMR (500 MHz, CHCl_3_-d): δ ppm 3.86 (d, *J* = 16.38 Hz, 1H, CH_2_), 4.10 (d, *J* = 16.63 Hz, 1H, CH_2_), 6.73 (s, 1H, N-CH-S), 7.26–7.40 (m, 4H, Ar-C17, C18, C20, C21), 7.56 (br d, *J* = 7.83 Hz, 1H, Ar-C5), 7.76 (br d, *J* = 8.80 Hz, 1H, Ar-C4), 8.13 (s, 1H, Ar-C7). ^13^C-NMR (500 MHz, CHCl_3_-d): δ ppm 32.84 (1C, CH_2_), 63.11 (1C, N-CH-S), 119.02, 120.82, 122.76, 127.98(2C), 129.74(2C), 131.97, 133.30, 135.46, 138.85, 148.06 (12C, Ar), 158.04 (1C, N=C-N), 171.19 (1C, C=O).

Synthesis of 3-(6-chlorobenzo[*d*]thiazol-2-yl)-2-(4-methoxyphenyl)thiazolidin-4-one (**18**).

Reaction time: 13 h (A). Yield: 62.3%. M p. 220–221 °C: Rf: 0.62 (petroleum ether/ethyl acetate: 8/2). IR: (cm^−1^, Nujol): 2364 (=Ν-), 1709 (C=O), 1571 (C=C arom), 1222 (-OCH_3_). ^1^H-NMR (500 MHz, CHCl_3_-d): δ ppm 3.76 (s, 3H, O-CH_3_), 3.91 (d, *J*=16.38 Hz, 1H, CH_2_), 4.10 (d, *J* = 16.63 Hz, 1H, CH_2_), 6.72 (s, 1H, N-CH-S), 6.82 (d, *J* = 8.80 Hz, 2H, Ar-C18, C20), 7.12 (t, *J* = 8.56 Hz, 1H, Ar-C5), 7.23–7.38 (m, 1H, Ar-C4), 7.46–7.53 (m, 2H, Ar-C17, C21), 8.08 (s, 1H, Ar-C7). ^13^C-NMR (500 MHz, CHCl_3_-d): δ ppm 32.88 (1C, CH_2_), 55.27 (1C, O-CH_3_), 63.47 (1C, N-CH-S), 114.20(2C), 120.79, 126.76, 126.79(2C), 127.73, 129.92, 132.28, 133.36, 147.44, 158.89 (12C, Ar), 163.59 (1C, N=C-N), 171.01 (1C, C=O).

Synthesis of (3-(6-chlorobenzo[*d*]thiazol-2-yl)-2-(4-hydroxyphenyl)thiazolidin-4-one (**19**).

Reaction time 18 h (A), 30 min, 100 °C (B), respectively. Yield: 24.1% (A), 72.5% (B) respectively. Mp. 236–237°C °C: Rf: 0.40 (petroleum ether/ethyl acetate: 8/2). IR: (cm^−1^, Nujol): 3198 (-OH), 2358 (=Ν-), 1709 (C=O), 1573 (C=C arom). ^1^H-NMR (500 MHz, CHCl_3_-d): δ ppm 3.86 (d, *J* = 16.38 Hz, 1H, CH_2_), 4.10 (d, *J* = 16.63 Hz, 1H, CH_2_), 5.35 (br s, 1H, -OH), 6.73 (s, 1H, N-CH-S), 7.26–7.43 (m, 5H, Ar-C5, C17, C18, C20, C21), 7.76 (br d, *J* = 8.80 Hz, 1H, Ar-C4), 8.13 (s, 1H, Ar-C7). ^13^C-NMR (500 MHz, CHCl_3_-d) δ ppm 33.12 (1C, CH_2_), 63.71 (1C, N-CH-S), 115.75 (2C), 118.23, 121.55, 125.14, 129.02, 130.01(2C), 131.52, 132.87, 151.68, 156.71 (12C, Ar), 163.77 (1C, N=C-N), 171.12 (1C, C=O). (MS): (m/z) 363 (M^+^, 100%), 288 (88%), 272 (9%), 225 (33%), 184 (48%), 146 (7%), 136 (21%), 115 (18%), 73 (17%).

### 3.2. Biological Evaluation

#### 3.2.1. Antibacterial Action

Bacterial strains utilized include Gram-negative: *Salmonella typhimurium*, (ATCC 13311).

*Pseudomonas aeruginosa* (ATCC 27853), *Escherichia coli* (ATCC 35210)*,* and Gram-positive *bacteria: Staphylococcus aureus* (ATCC 6538) and *Listeria monocytogenes* (NCTC 7973) bacteria. Pathogens were provided from the Mycological Laboratory, Institute for Biological Research “Siniša Stankovic” Belgrade. Resistant strains used were MRSA IBRS MRSA 011, *E. coli* IBRS E003 and *P. aeruginosa* IBRS P001 obtained as described in Kartsev et al. [62]. The MIC/MBC were effectuated utilizing microdilution assay as previous described [63,64].

##### *E.* *coli*

Sensitivity studies of *E. coli* strain were tested by the disc diffusion method on Mueller Hinton agar with the use of antibiogram discs (Bioanalyse) and tablets (Torlak, Serbia) for the following antibiotics: penicillin, amoxicillin, tetracycline, neomicin, gentamicin, colistin, ceftriaxon, sulfamethaxasole with trimetoprim, enrofloxacin and florfenicol*. E. coli* strain was resistant to all tested antibiotics with the exception of enrofloxacin, colistin and florfenicol [65]. It is described in detail in our previous paper [46].

##### *Pseudomonas* *aeruginosa*

Sensitivity studies of *P. aeruginosa* strain were tested by the disc diffusion method on Mueller Hinton agar with the use of antibiogram discs (Bioanalyse) and tablets (Torlak, Serbia) for the following antibiotics: penicillin, amoxicillin, tetracycline, neomicin, gentamicin, ceftriaxon, sulfamethaxasole with trimetoprim, enrofloxacin and florfenicol*. P. aeruginosa* strain was resistant to all tested antibiotics with the exception of enrofloxacin, and florfenicol [65,66].

#### 3.2.2. Inhibition of Biofilm Formation

This method was performed as described previously [48,67] with some modifications. The percentage of inhibition of biofilm formation was calculated by the following formula:

[(A_620_ control − A_620_ sample)/A_620_ control] × 100 (1)

#### 3.2.3. Antifungal Activity

For the antifungal bioassays, six fungi were used: *Aspergillus niger* (ATCC 6275), *Aspergillus fumigatus* (human isolate), *Aspergillus versicolor* (ATCC 11730), *Penicillium funiculosum* (ATCC 36839), *Trichoderma viride* (IAM 5061), *Penicillium verrucosum* var. *cyclopium* (food isolate). The organisms were obtained from the Mycological Laboratory, Department of Plant Physiology, Institute for Biological Research ‘Siniša Stankovic’, Belgrade, Serbia. All experiments were performed in duplicate and repeated three times [68,69].

### 3.3. Statistical Analysis

All tests were performed three times and the values were determined as standard deviation (SD) and mean values. One-way ANOVA test was allowed to determine variance analysis with Tukey HSD Test (0.05 levels). Analysis was executed with the help of SPSS statistics software (version 18).

### 3.4. Drug-Likeness

The targeted molecules were appraised for predicting the Drug-likeness based on 5 separate filters namely Egan [55], Ghose [53], Muegge [56], Veber [54] and Lipinski [52] rules accompanying bioavailability and Drug-likeness scores using the Molsoft software and SwissADME program (http://swissadme.ch, accessed on 28 June 2021) using the ChemAxon’s Marvin JS structure drawing tool.

### 3.5. Docking Studies

Protein Preparation: X-ray crystal structures of *E. coli* DNA GyrB, Thymidylate kinase, *E. coli* primase, *E. coli* MurB, LD-carboxypeptidase, (PDB code: 1KZN, AQGG, 1DDE, 2Q85, 1ZRS, respectively) were retrieved from Brookhaven Protein Data Bank (PDB). The pdb files of proteins were submitted to “Build/check/repair model” to the session “Prepare PDB file for docking programs” and missing side chains were modeled in; water positions and symmetry were corrected, and hydrogen atoms were added. Only chain A of each enzyme of the repaired pdb file was evaluated and passed to AutodockTools (ADT ver. 1.5.6) for pdbqt file preparation. ADT assigned polar hydrogens, water molecules and non-standard residues were removed, only polar hydrogens were maintained, and Gasteiger charges were computed for protein atoms. AutoDock saved the prepared file in PDBQT format.

Ligand Preparation: All the molecules were sketched in chemdraw12.0 program. The geometry of built compounds was optimized using the molecular mechanical force fields 94 (MMFF94) energy via program LigandScout, partial charges were also calculated, comformers of each ligand were generated and the best one was maintained and saved as mol2 files that were passed to ADT for pdbqt file preparation. Polar hydrogens were added to each structure, followed by computing Gasteiger and Kollman charges, and the torsions.

Docking Procedure: Autodock 4 (ver. 4.2.6) was employed for docking simulations. The region of interest, used by Autodock4 for docking runs and by Autogrid4 for affinity grid maps preparation, was defined in such a way to comprise the whole catalytic binding site using a grid of 50 × 50 × 50 points with a grid space of 0.375 Å. All parameters used in docking were default. The translation, quaternion and torsions steps were taken from default values in AutoDock. The Lamarckian genetic algorithm and the pseudo-Solis and Wets methods were applied for minimization using default parameters. The number of docking runs was 100. After docking, the 100 solutions were clustered into groups with RMS lower than 1.0 Ε. The clusters were ranked by the lowest energy representative of each cluster. Upon completion of docking, the best poses were screened by examination of binding energy (Δ*G*_binding_, kcal/mol) and number in cluster. A preliminary blind docking was performed in order to validate the protocol. The RMSD values were predicted by superimposing each docked co-ligand on its original crystallographic bound conformation. The RMSD of all enzymes were in range of 0.85 to 1.43, which are acceptable.

In order to describe the ligand-binding pocket interactions, the top ranked binding mode found by AutoDock in complex with the binding pocket of each enzyme was selected. The resulting poses and potential interactions were visualized using the Discovery studio visualizer version 4.0 (BIOVIA, San Diego, CA, USA) and ligPlot+ (ver. 2.2).

## 4. Conclusions

With the purpose to improve the antibacterial activity of previously synthesized compounds, modifications were performed to the initial structure and nineteen new derivatives with 6-Cl, 4-OMe, 6-CN, 6-adamantan, 4-Me,6-adamantan substituents at benzothiazole ring were synthesized and evaluated in silico and experimentally for their antimicrobial activity against panel of four bacterial strains *S. aureus, L. monocytogenes, E. coli* and *S. Typhimirium* and three resistant strains MRSA, *E. coli* and *P. aeruginosa.*

It was observed that among all compounds tested the derivatives of 6-chlorobenzothiazole-based thiazolidinone exhibited higher activity, with compound **18** being the most promising (MIC and MBC at 0.10–0.25 mg/mL and 0.12–0.5 mg/mL, respectively). It should be mentioned that some of compounds exhibited superior/equal activity mostly against *P. aeruginosa* resistant and non-resistant*, E. coli* and two of them against the most resistant *L. monocytogenes* than reference drugs, ampicillin and streptomycin. The most sensitive bacteria appeared to be *E. coli.*

Compounds with the most promising antibacterial potential were studied for their effect on biofilm formation. It was found that the activity of tested compounds in concentration of MIC did not exceed the activity of reference drugs. On the other hand, in concentration of 0.5 MIC compound **19** exhibited higher antibiofilm activity than streptomycin. The comparison of obtained results on antibacterial activity with those of compounds with 6-OCF_3_ substituent in benzothiazole ring revealed that activity depends on substitutients not only at benzothiazole moiety but also of benzene ring. Thus, in case of 4-F substitution at benzene ring the replacement of 6-OCF_3_ by 6-Cl improved twice the activity against *P. aeruginosa* and *P. aeruginosa* resistant, while the replacement by 4-OMe slightly improve only activity against *E. coli* (0.15 mg/mL and 0.12mg/mL). Among 4-nitro derivatives the presence of 6-Cl as well as 6-CN substituent at benzothiazole ring appeared to be beneficial compared to 6-OCF_3_ and 4-OCH_3,_ since activity against *S. aureus*, MRSA and resistant strain of *E. coli* increased 2.5 folds, while against *L. monocytogenes* and *S. typhimurium* from 4- to 5-folds better results (2–3 times) were obtained in case of the presence of 4-OMe and 4-OH substituents in benzene ring of 6-Cl-benzothiazole derivatives compared to the same substituents at the 4 position of benzene ring of 6-OCF_3_ derivatives.

Docking analysis to DNA Gyrase, thymidylate kinase, *E. coli* primase, *E. coli* MurB and *E. coli* LD carboxypeptidase indicate the probable involvement of the last enzyme in the mechanism of the antibacterial activity of the tested compounds.

Antifungal activity was moderate to low, lower than antibacterial. Finally, it can be concluded that in general compounds **15**–**19** and especially **18** are promising for further modifications in order to develop new more active antibacterial agents.

## Figures and Tables

**Table 1:** Predicted toxicity with program PROTOX.

No.	Predicted LD50	Predicted Toxicity Class	Hepatotoxicity	Carcinogenicity	Immunotoxicity	Mutagenicity	Cytotoxicity
**1**	500 mg/kg	4	Inactive 0.54	Inactive 0.57	Inactive 0.99	Inactive 0.67	Inactive 0.76
**2**	1000 mg/kg	4	Inactive 0.52	Inactive 0.63	Inactive 0.97	Inactive 0.66	Inactive 0.62
**3**	500 mg/kg	4	Inactive 0.56	Inactive 0.65	Inactive 0.99	Inactive 0.67	Inactive 0.78
**4**	500 mg/kg	4	Inactive 0.54	Inactive 0.57	Inactive 0.99	Inactive 0.67	Inactive 0.76
**5**	500 mg/kg	4	Inactive 0.58	Inactive 0.56	Inactive 0.99	Inactive 0.66	Inactive 0.77
**6**	500 mg/kg	4	Inactive 0.50	Inactive 0.55	Inactive 0.94	Inactive 0.64	Inactive 0.75
**7**	500 mg/kg	4	Inactive 0.52	Inactive 0.55	Inactive 0.79	Inactive 0.64	Inactive 0.74
**8**	500 mg/kg	4	Inactive 0.52	Inactive 0.56	Inactive 0.96	Inactive 0.63	Inactive 0.75
**9**	500 mg/kg	4	Inactive 0.54	Inactive 0.56	Inactive 0.94	Inactive 0.63	Inactive 0.77
**10**	500 mg/kg	4	Inactive 0.50	Inactive 0.62	Inactive 0.98	Inactive 0.61	Inactive 0.63
**11**	500 mg/kg	4	Inactive 0.52	Inactive 0.64	Inactive 0.99	Inactive 0.60	Inactive 0.63
**12**	500 mg/kg	4	Inactive 0.59	Inactive 0.60	Inactive 0.98	Inactive 0.66	Inactive 0.66
**13**	1000 mg/kg	4	Inactive 0.52	Inactive 0.65	Inactive 0.99	Inactive 0.66	Inactive 0.74
**14**	500 mg/kg	4	Inactive 0.56	Inactive 0.60	Inactive 0.98	Inactive 0.67	Inactive 0.65
**15**	500 mg/kg	4	Inactive 0.57	Inactive 0.55	Inactive 0.99	Inactive 0.67	Inactive 0.81
**16**	500 mg/kg	4	Inactive 0.60	Inactive 0.58	Inactive 0.99	Inactive 0.68	Inactive 0.68
**17**	500 mg/kg	4	Inactive 0.57	Inactive 0.55	Inactive 0.99	Inactive 0.67	Inactive 0.81
**18**	1000 mg/kg	4	Inactive 0.52	Inactive 0.65	Inactive 0.99	Active 0.91	Inactive 0.74
**19**	500 mg/kg	4	Inactive 0.54	Inactive 0.55	Inactive 0.99	Inactive 0.68	Inactive 0.82

**Table 2:** Prediction of carcinogenicity and mutagenesis of compounds by ToxPredict application.

N	Carcinogenicity (Rodents (Multiple Species/Sites))	Carcinogenicity (Rat)	Carcinogenicity (Mouse)	Mutagenicity (*Salmonella typhimurium*)
**1**	Inactive Conf.: 0.026	Inactive Conf.: 0.026	Inactive Conf.: 0.026	Inactive Conf.: 0.026
**2**	Inactive Conf.: 0.027	Inactive Conf.: 0.027	Inactive Conf.: 0.026	Inactive Conf.: 0.027
**3**	Inactive Conf.: 0.024	Inactive Conf.: 0.027	Inactive Conf.: 0.033	Inactive Conf.: 0.036
**4**	Inactive Conf.: 0.031	Inactive Conf.: 0.028	Inactive Conf.: 0.041	Inactive Conf.: 0.028
**5**	Inactive Conf.: 0.028	Inactive Conf.: 0.028	Inactive Conf.: 0.041	Inactive Conf.: 0.026
**6**	Inactive Conf.: 0.026	Inactive Conf.: 0.027	Inactive Conf.: 0.029	Inactive Conf.: 0.026
**7**	Inactive Conf.: 0.031	Inactive Conf.: 0.027	Inactive Conf.: 0.031	Inactive Conf.: 0.028
**8**	Inactive Conf.: 0.025	Inactive Conf.: 0.028	Inactive Conf.: 0.027	Inactive Conf.: 0.028
**9**	Inactive Conf.: 0.028	Inactive Conf.: 0.026	Inactive Conf.: 0.049	Inactive Conf.: 0.041
**10**	Inactive Conf.: 0.033	Inactive Conf.: 0.028	Inactive Conf.: 0.041	Inactive Conf.: 0.031
**11**	Inactive Conf.: 0.032	Inactive Conf.: 0.026	Inactive Conf.: 0.041	Inactive Conf.: 0.033
**12**	Inactive Conf.: 0.028	Inactive Conf.: 0.029	Inactive Conf.: 0.028	Inactive Conf.: 0.028
**13**	Inactive Conf.: 0.031	Inactive Conf.: 0.028	Inactive Conf.: 0.041	Inactive Conf.: 0.039
**14**	Inactive Conf.: 0.034	Inactive Conf.: 0.028	Inactive Conf.: 0.041	Inactive Conf.: 0.038
**15**	Inactive Conf.: 0.026	Inactive Conf.: 0.026	Inactive Conf.: 0.026	Inactive Conf.: 0.028
**16**	Inactive Conf.: 0.026	Inactive Conf.: 0.026	Inactive Conf.: 0.026	Inactive Conf.: 0.026
**17**	Inactive Conf.: 0.028	Inactive Conf.: 0.028	Inactive Conf.: 0.029	Inactive Conf.: 0.027
**18**	Inactive Conf.: 0.026	Inactive Conf.: 0.027	Inactive Conf.: 0.033	Inactive Conf.: 0.035
**19**	Inactive Conf.: 0.033	Inactive Conf.: 0.027	Inactive Conf.: 0.041	Inactive Conf.: 0.027

**Table 3:** Antibacterial activity of compounds (MIC and MBC in mg/mL).

Compounds		*S.a.*	MRSA	*L.m.*	*P.a.*	*P.a.* Res	*E. coli*	*E. coli* Res	*S. ty.*
**1**	MIC	0.30 ± 0.07	0.50 ± 0.00	0.50 ± 0.00	0.25 ± 0.00	0.25 ± 0.11	0.12 ± 0.00	0.25 ± 0.11	0.50 ± 0.00
	MBC	0.50 ± 0.00	1.00 ± 0.00	0.83 ± 0.11	0.58 ± 0.11	0.50 ± 0.00	0.25 ± 0.00	0.50 ± 0.00	0.75 ± 0.05
**2**	MIC	0.12 ± 0.00	0.25 ± 0.00	0.25 ± 0.00	0.30 ± 0.07	0.25 ± 0.04	0.12 ± 0.00	0.25 ± 0.00	0.25 ± 0.04
	MBC	0.25 ± 0.00	0.50 ± 0.00	0.75 ± 0.00	0.50 ± 0.00	0.50 ± 0.00	0.25 ± 0.00	0.50 ± 0.00	0.50 ± 0.00
**3**	MIC	0.50 ± 0.00	0.30 ± 0.07	0.50 ± 0.00	0.25 ± 0.04	0.20 ± 0.01	0.20 ± 0.00	0.25 ± 0.00	0.30 ± 0.07
	MBC	1.00 ± 0.00	0.50 ± 0.00	1.00 ± 0.00	0.50 ± 0.00	0.25 ± 0.04	0.25 ± 0.04	0.50 ± 0.00	0.50 ± 0.00
**4**	MIC	0.25 ± 0.04	0.25 ± 0.00	0.25 ± 0.00	0.25 ± 0.00	0.20 ± 0.01	0.10 ± 0.00	0.25 ± 0.04	0.25 ± 0.00
	MBC	0.50 ± 0.00	0.50 ± 0.00	0.50 ± 0.00	0.50 ± 0.00	0.25 ± 0.04	0.12 ± 0.00	0.50 ± 0.00	0.83 ± 0.11
**5**	MIC	0.25 ± 0.00	0.30 ± 0.07	0.50 ± 0.00	0.25 ± 0.04	0.12 ± 0.00	0.12 ± 0.00	0.25 ± 0.00	0.50 ± 0.00
	MBC	0.50 ± 0.00	0.50 ± 0.00	1.00 ± 0.00	0.50 ± 0.00	0.25 ± 0.00	0.25 ± 0.00	0.50 ± 0.00	1.00 ± 0.00
**6**	MIC	0.12 ± 0.00	0.25 ± 0.00	0.25 ± 0.00	0.25 ± 0.04	0.20 ± 0.00	0.25 ± 0.00	0.25 ± 0.00	0.50 ± 0.00
	MBC	0.25 ± 0.04	0.50 ± 0.00	0.50 ± 0.00	0.50 ± 0.00	0.25 ± 0.04	0.50 ± 0.00	0.50 ± 0.00	0.83 ± 0.11
**7**	MIC	0.20 ± 0.00	0.50 ± 0.00	0.75 ± 0.00	0.25 ± 0.04	0.25 ± 0.00	0.20 ± 0.00	0.25 ± 0.00	0.50 ± 0.00
	MBC	0.25 ± 0.04	1.00 ± 0.00	1.00 ± 0.00	0.58 ± 0.11	0.50 ± 0.00	0.25 ± 0.04	0.50 ± 0.00	0.83 ± 0.11
**8**	MIC	0.20 ± 0.00	0.50 ± 0.00	0.30 ± 0.07	0.25 ± 0.00	0.06 ± 0.00	0.20 ± 0.00	0.25 ± 0.00	0.30 ± 0.07
	MBC	0.25 ± 0.04	1.00 ± 0.00	0.50 ± 0.00	0.50 ± 0.00	0.12 ± 0.00	0.25 ± 0.004	0.50 ± 0.00	0.50 ± 0.00
**9**	MIC	0.12 ± 0.00	0.58 ± 0.11	0.50 ± 0.00	0.25 ± 0.04	0.30 ± 0.07	0.20 ± 0.00	0.25 ± 0.00	0.50 ± 0.00
	MBC	0.25 ± 0.04	1.00 ± 0.00	1.00 ± 0.00	0.50 ± 0.00	0.50 ± 0.00	0.25 ± 0.04	0.50 ± 0.00	1.00 ± 0.00
**Streptomycin**	MIC	0.10 ± 0.00	0.10 ± 0.00	0.15 ± 0.00	0.10 ± 0.00	0.05 ± 0.00	0.10 ± 0.00	0.10 ± 0.00	0.10 ± 0.00
	MBC	0.20 ± 0.01		0.30 ± 0.01	0.20 ± 0.01	0.10 ± 0.00	0.20 ± 0.00	0.20 ± 0.01	0.20 ± 0.01
**Ampicillin**	MIC	0.10 ± 0.00		0.15 ± 0.00	0.30 ± 0.01	0.2 ± 0.01	0.15 ± 0.00	0.20 ± 0.01	0.10 ± 0.00
	MBC	0.15 ± 0.00		0.30 ± 0.02	0.50 ± 0,01		0.20 ± 0.01		0.20 ± 0.00

MIC—minimal inhibitory concentration, MBC—minimal bactericidal concentration, *S.a*.—*S. aurues* (ATCC 6538), *l.m*.—*L. monocytogenes* (NCTC 7973), *P.a*.—*P. aeruginosa* (ATCC 27853), *S. ty.—S. typhimirium* (ATCC 13311), MRSA—methicillin resistant *S. aureus*, (IBRS MRSA 011), *E. coli* res—resistant *E. coli* (IBRS E003), *P.a.* res—resistant *P. aeruginosa* (IBRS P001).

**Table 4:** Antibacterial activity of compounds **10**–**19** (MIC and MBC in mg/mL).

Compounds		*S.a.*	MRSA	*L.m.*	*P.a.*	*P.a.* Res	*E. coli*	*E. coli* Res	*S. ty.*
**10**	MIC	0.50 ± 0.00	0.75 ± 0.00	0.75 ± 0.02	0.20 ± 0.00	0.12 ± 0.00	0.12 ± 0.00	0.50 ± 0.03	0.50 ± 0.02
	MBC	1.00 ± 0.08	1.00 ± 0.00	>1.00	0.25 ± 0.00	0.25 ± 0.00	0.25 ± 0.00	1.00 ± 0.04	1.00 ± 0.04
**11**	MIC	0.50 ± 0.02	0.75 ± 0.03	0.50 ± 0.02	0.25 ± 0.00	0.15 ± 0.00	0.25 ± 0.00	0.50 ± 0.03	0.50 ± 0.02
	MBC	0.75 ± 0.04	1.00 ± 0.06	1.00 ± 0.06	0.50 ± 0.03	0.25 ± 0.01	0.50 ± 0.03	0.75 ± 0.02	1.00 ± 0.04
**12**	MIC	0.50 ± 0.02	0.75 ± 0.03	0.50 ± 0.03	0.25 ± 0.01	0.25 ± 0.00	0.25 ± 0.01	0.50 ± 0.03	0.50 ± 0.01
	MBC	1.00 ± 0.06	>1.00	>1.00	0.50 ± 0.03	0.50 ± 0.02	0.50 ± 0.02	1.00 ± 0.08	1.00 ± 0.06
**13**	MIC	0.50 ± 0.02	0.25 ± 0.00	0.75 ± 0.04	0.30 ± 0.01	0.15 ± 0.00	0.12 ± 0.00	0.25 ± 0.00	0.50 ± 0.03
	MBC	0.75 ± 0.04	0.50 ± 0.00	1.00 ± 0.08	0.50 ± 0.02	0.25 ± 0.01	0.25 ± 0.01	0.50 ± 0.03	1.00 ± 0.04
**14**	MIC	0.25 ± 0.01	0.75 ± 0.03	0.50 ± 0.02	0.25 ± 0.01	0.15 ± 0.00	0.12 ± 0.00	0.25 ± 0.00	0.12 ± 0.00
	MBC	0.50 ± 0.02	1.00 ± 0.06	1.00 ± 0.08	0.50 ± 0.03	0.25 ± 0.01	0.25 ± 0.01	0.50 ± 0.03	0.50 ± 0.02
**15**	MIC	0.30 ± 0.01	0.50 ± 0.03	0.25 ± 0.01	0.12 ± 0.00	0.12 ± 0.00	0.25 ± 0.01	0.25 ± 0.00	0.50 ± 0.03
	MBC	0.50 ± 0.03	0.75 ± 0.00	0.50 ± 0.03	0.25 ± 0.00	0.25 ± 0.00	0.50 ± 0.02	0.50 ± 0.00	0.75 ± 0.03
**16**	MIC	0.10 ± 0.00	0.10 ± 0.00	0.06 ± 0.00	0.30 ± 0.00	0.25 ± 0.01	0.30 ± 0.01	0.12 ± 0.00	0.10 ± 0.00
	MBC	0.12 ± 0.00	0.12 ± 0.00	0.12 ± 0.00	0.60 ± 0.00	0.50 ± 0.03	0.60 ± 0.03	0.25 ± 0.00	0.12 ± 0.00
**17**	MIC	0.50 ± 0.02	0.50 ± 0.02	0.25 ± 0.00	0.12 ± 0.00	0.10 ± 0.00	0.25 ± 0.01	0.25 ± 0.00	0.30 ± 0.00
	MBC	0.75 ± 0.03	0.75 ± 0.04	0.50 ± 0.02	0.25 ± 0.01	0.12 ± 0.00	0.50 ± 0.02	0.50 ± 0.03	0.50 ± 0.03
**18**	MIC	0.20 ± 0.00	0.20 ± 0.00	0.12 ± 0.00	0.10 ± 0.00	0.06 ± 0.00	0.12 ± 0.00	0.25 ± 0.01	0.25 ± 0.00
	MBC	0.25 ± 0.01	0.25 ± 0.00	0.20 ± 0.00	0.12 ± 0.00	0.12 ± 0.00	0.20 ± 0.01	0.50 ± 0.02	0.50 ± 0.02
**19**	MIC	0.25 ± 0.01	0.25 ± 0.00	0.12 ± 0.00	0.12 ± 0.00	0.10 ± 0.00	0.12 ± 0.00	0.12 ± 0.00	0.25 ± 0.00
	MBC	0.50 ± 0.02	0.50 ± 0.02	0.25 ± 0.00	0.25 ± 0.00	0.12 ± 0.00	0.25 ± 0.01	0.25 ± 0.00	0.50 ± 0.02
**Streptomycin**	MIC	0.10 ± 0.00	0.10 ± 0.00	0.15 ± 0.00	0.10 ± 0.00	0.05 ± 0.00	0.10 ± 0.00	0.10 ± 0.00	0.10 ± 0.00
	MBC	0.20 ± 0.01		0.30 ± 0.01	0.20 ± 0.01	0.10 ± 0.00	0.20 ± 0.00	0.20 ± 0.01	0.20 ± 0.01
**Ampicillin**	MIC	0.10 ± 0.00		0.15 ± 0.00	0.30 ± 0.01	0.2 ± 0.01	0.15 ± 0.00	0.20 ± 0.01	0.10 ± 0.00
	MBC	0.15 ± 0.00		0.30 ± 0.02	0.50 ± 0,01		0.20 ± 0.01		0.20 ± 0.00

MIC—minimal inhibitory concentration, MBC—minimal bactericidal concentration, *S.a*.—*S. aurues* (ATCC 6538), *L.m*.—*L. monocytogenes* (NCTC 7973), *P.a*.—*P. aeruginosa* (ATCC 27853), *S. ty*.—*S. typhimurium* (ATCC 13311), MRSA—Methicillin resistant *S. aureus* (IBRS MRSA 011), *E. coli* res—resistant *E. coli* (IBRS E003), *P.a.* res—*P. aeruginosa* (IBRS P001).

**Table 5:** Inhibition of *P. aeruginosa* biofilm formation (%).

Compound	MIC	0.5 MIC
**16**	49.46 ± 5.67	35.55 ± 2.30
**18**	NE	NE
**19**	51.46 ± 8.76	41.76 ± 9.99
**8**	NE	NE
**Ampicillin**	70.00 ± 10.23	52.36 ± 3.67
**Streptomycin**	63.56 ± 8.28	29.12 ± 1.22

NE—no effect.

**Table 6:** Antifungal activity of tested compounds (MIC and MBC in mg/mL).

Compounds		*A.f.*	*A.n.*	*A.v.*	*P.f.*	*T.v.*	*P.v.c.*
**1**	MIC	>1.00	0.50 ± 0.00	0.50 ± 0.00	0.50 ± 0.02	0.50 ± 0.00	0.50 ± 0.02
	MFC	>1.00	1.00 ± 0.00	1.00 ± 0.00	1.00 ± 0.00	1.00 ± 0.00	1.00 ± 0.00
**2**	MIC	>1.00	0.50 ± 0.00	1.00 ± 0.00	0.50 ± 0.00	0.50 ± 0.00	1.00 ± 0.00
	MFC	>1.00	1.00 ± 0.00	>1.00	1.00 ± 0.00	1.00 ± 0.00	>1.00
**3**	MIC	>1.00	1.00 ± 0.00	1.00 ± 0.00	0.50 ± 0.00	0.50 ± 0.02	0.50 ± 0.00
	MFC	>1.00	>1.00	>1.00	1.00 ± 0.00	1.00 ± 0.00	1.00 ± 0.00
**4**	MIC	1.00 ± 0.00	0.50 ± 0.02	0.50 ± 0.02	0.50 ± 0.02	1.00 ± 0.00	>1.00
	MFC	>1.00	1.00 ± 0.00	1.00 ± 0.00	1.00 ± 0.00	>1.00	>1.00
**5**	MIC	>1.00	>1.00	>1.00	>1.00	>1.00	>1.00
	MFC	>1.00	>1.00	>1.00	>1.00	>1.00	>1.00
**6**	MIC	>1.00	>1.00	0.25 ± 0.00	0.25 ± 0.04	0.25 ± 0.04	0.25 ± 0.04
	MFC	>1.00	>1.00	0.50 ± 0.02	0.50 ± 0.02	0.50 ± 0.02	0.50 ± 0.02
**7**	MIC	0.50 ± 0.00	1.00 ± 0.00	0.25 ± 0.04	0.50 ± 0.00	0.50 ± 0.00	0.50 ± 0.00
	MFC	1.00 ± 0.00	>1.00	0.50 ± 0.00	1.00 ± 0.00	1.00 ± 0.00	1.00 ± 0.00
**8**	MIC	>1.00	>1.00	0.50 ± 0.00	0.50 ± 0.00	0.50 ± 0.00	0.50 ± 0.00
	MFC	>1.00	>1.00	1.00 ± 0.00	1.00 ± 0.00	1.00 ± 0.00	1.00 ± 0.00
**9**	MIC	0.50 ± 0.02	1.00 ± 0.00	0.25 ± 0.00	0.50 ± 0.00	0.25 ± 0.00	0.50 ± 0.00
	MFC	1.00 ± 0.00	>1.00	0.50 ± 0.00	1.00 ± 0.00	0.50 ± 0.00	0.83 ± 0.11
**10**	MIC	0.50 ± 0.02	1.00 ± 0.00	0.25 ± 0.00	1.00 ± 0.00	0.50 ± 0.02	0.50 ± 0.02
	MBC	1.00 ± 0.00	>1.00	0.50 ± 0.03	>1.00	1.00 ± 0.00	1.00 ± 0.00
**11**	MIC	1.00 ± 0.00	>1.00	0.50 ± 0.01	0.50 ± 0.03	0.25 ± 0.00	0.50 ± 0.00
	MBC	>1.00	>1.00	1.00 ± 0.00	1.00 ± 0.00	0.50 ± 0.02	1.00 ± 0.00
**12**	MIC	0.50 ± 0.02	1.00 ± 0.00	0.50 ± 0.02	1.00 ± 0.00	0.50 ± 0.02	0.50 ± 0.02
	MBC	1.00 ± 0.00	>1.00	1.00 ± 0.00	>1.00	1.00 ± 0.00	1.00 ± 0.00
**13**	MIC	1.00 ± 0.00	1.00 ± 0.00	0.50 ± 0.02	0.50 ± 0.02	0.50 ± 0.03	0.50 ± 0.03
	MBC	>1.00	>1.00	1.00 ± 0.00	1.00 ± 0.00	1.00 ± 0.00	1.00 ± 0.00
**14**	MIC	1.00 ± 0.00	0.50 ± 0.03	0.25 ± 0.00	0.50 ± 0.03	0.50 ± 0.03	0.50 ± 0.02
	MBC	>1.00	1.00 ± 0.00	0.50 ± 0.00	1.00 ± 0.00	1.00 ± 0.00	1.00 ± 0.00
**15**	MIC	1.00 ± 0.00	>1.00	1.00 ± 0.00	0.50 ± 0.00	0.50 ± 0.02	0.25 ± 0.00
	MBC	>1.00	>1.00	>1.00	1.00 ± 0.00	1.00 ± 0.00	0.50 ± 0.02
**16**	MIC	0.12 ± 0.00	1.00 ± 0.00	0.50 ± 0.00	0.50 ± 0.02	0.50 ± 0.02	1.00 ± 0.00
	MBC	0.25 ± 0.00	>1.00	1.00 ± 0.00	1.00 ± 0.00	1.00 ± 0.00	>1.00
**17**	MIC	1.00 ± 0.00	>1.00	0.50 ± 0.02	0.50 ± 0.02	0.25 ± 0.00	0.25 ± 0.00
	MBC	>1.00	>1.00	1.00 ± 0.00	1.00 ± 0.00	0.50 ± 0.02	0.50 ± 0.00
**18**	MIC	0.50 ± 0.01	0.50 ± 0.02	0.12 ± 0.00	0.50 ± 0.02	0.25 ± 0.00	0.25 ± 0.04
	MBC	1.00 ± 0.00	1.00 ± 0.00	0.25 ± 0.00	1.00 ± 0.00	0.50 ± 0.02	0.50 ± 0.02
**19**	MIC	0.50 ± 0.03	0.50 ± 0.00	0.25 ± 0.04	0.25 ± 0.00	0.25 ± 0.00	0.12 ± 0.00
	MBC	1.00 ± 0.00	1.00 ± 0.00	0.50 ± 0.02	0.50 ± 0.02	0.50 ± 0.02	0.25 ± 0.01
**Bifonazole**	MIC	1.00 ± 0.00	>1.00	1.00 ± 0.00	0.50 ± 0.00	0.50 ± 0.02	0.25 ± 0.00
	MFC	0.20 ± 0.000	0.20 ± 0.000	0.20 ± 0.01	0.25 ± 0.01	0.20 ± 0.01	0.20 ± 0.00
**Ketoconazole**	MIC	0.20 ± 0.01	0.20 ± 0.01	0.20 ± 0.00	0.20 ± 0.00	1.00 ± 0.01	0.20 ± 0.01
	MFC	0.50 ± 0.03	0.50 ± 0.03	0.50 ± 0.02	0.50 ± 0.03	1.50 ± 0.02	0.30 ± 0.01

*A.v*.—*A. versicolor* (ATCC 11730), *T.v*.—*T. viride* (IAM 5061), *A.n*.—*A. niger* (ATCC 6275), *P.v.c*.—P*enicillium verrucosum* var. *cyclopium* (food isolate). *P.f*.—*P. funiculosum* (ATCC 36839), *A.f*.—*A. fumigatus* (human isolate).

**Table 7:** Drug likeness predictions and physicochemical-pharmacokinetic/ADME properties of tested compounds.

No.	MW	Number of HBA ^a^	Number of HBD ^b^	Log *P*_o/w_^c^	Log S ^d^	TPSA ^e^	Absorption% ^f^	Lipinski, Ghose, Veber, Egan, and Muegge Violations	Bioavailability Score	Drug-Likeness Model Score
**1**	382.42	5	0	2.09	Moderately soluble	156.35	68.9	0	0.55	−0.45
**2**	373.4	5	0	2.7	Poorly soluble	110.53	55.1	0	0.55	−0.49
**3**	389.85	4	0	2.79	Poorly soluble	110.53	70.9	0	0.55	−0.42
**4**	406.31	3	0	2.87	Poorly soluble	110.53	70.9	0	0.55	−0.39
**5**	355.41	4	0	2.62	Moderately soluble	110.53	70.9	0	0.55	−0.07
**6**	515.52	2	0	4.3	Poorly soluble	86.74	79.1	2	0.17	0.22
**7**	499.06	3	0	4.24	Poorly soluble	86.74	79.1	1	0.55	0.2
**8**	529.54	2	0	4.55	Poorly soluble	86.74	79.1	2	0.17	0.56
**9**	496.63	4	0	4.34	Poorly soluble	86.74	79.1	1	0.55	0.45
**10**	360.43	4	0	3.02	Moderately soluble	95.97	76.4	0	0.55	0.28
**11**	387.43	5	0	2.49	Moderately soluble	141.79	59.1	0	0.55	−0.17
**12**	376.88	3	0	3.1	Poorly soluble	95.97	76.4	0	0.55	0.39
**13**	372.46	4	0	3.45	Moderately soluble	105.2	72.7	0	0.55	−0.06
**14**	358.43	4	1	2.56	Moderately soluble	116.2	No	0	0.55	0.13
**15**	364.84	3	0	3.25	Poorly soluble	86.74	79.1	0	0.55	−0.08
**16**	391.85	4	0	2.38	Moderately soluble	132.56	63.3	0	0.55	−0.13
**17**	381.3	2	0	3.42	Poorly soluble	86.74	79.1	0	0.55	−0.08
**18**	376.88	3	0	3.37	Poorly soluble	95.97	76.4	0	0.55	0.18
**19**	362.85	3	1	2.73	Moderately soluble	106.97	72.1	0	0.55	0.1

^a^ number of hydrogen bond acceptors; ^b^ number of hydrogen bond donors; ^c^ lipophilicity; ^d^ Water solubility (SILICOS-IT (S = soluble)); ^e^ topological polar surface area (Å^2^); ^f^ blood brain barrier permeant.

**Table 8:** Drug-likeness modes of tested compounds.

No.	Drug-Likeness Model	No.	Drug-Likeness Model	No.	Drug-Likeness Model
**1**	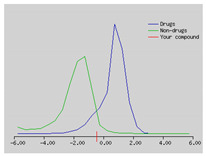	**8**	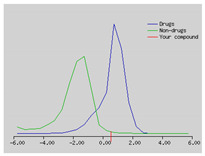	**14**	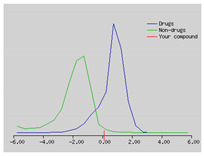
**2**	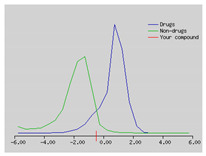	**9**	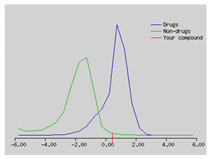	**15**	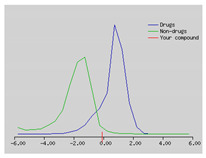
**3**	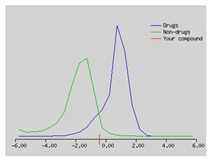	**10**	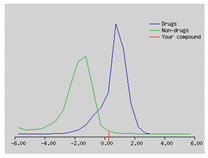	**16**	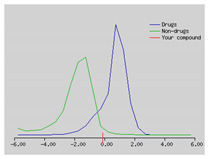
**4**	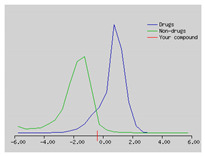	**11**	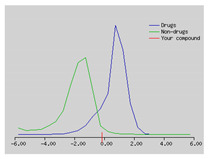	**17**	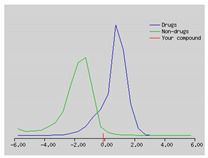
**5**	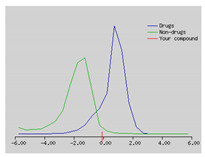	**12**	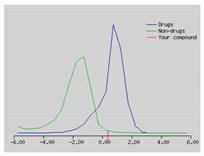	**18**	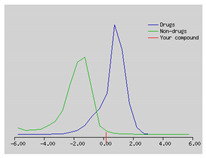
**6**	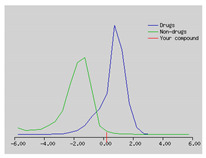	**13**	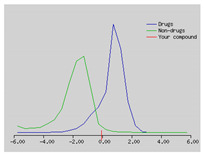	**19**	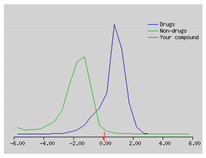
**7**	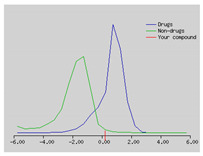				

**Table 9:** Molecular docking estimated free energy of binding to antibacterial targets.

No.	Est. Binding Energy (kcal/mol)						I-H LdcA	Residues Involved in H-Bond Formation LdcA
	*E. coli* Gyrase 1KZN	Thymidylate Kinase 4QGG	*E. coli* Primase 1DDE	*E. coli* MurB 2Q85	*E. coli* LdcA 1ZRS (R+)	*E. coli* LdcA 1ZRS (S−)		
**1**	-5.73(R+)	−2.41(S−)		−5.12(R+)	−5.81	−4.63		
**2**	−5.01(S−)	−1.24(S−)	−1.88(R+)	−5.96(R+)	−7.53	−7.23	2	Tyr138, **His285**
**3**	−4.12(R+)	−3.32(R+)		−5.23(S−)	−5.34	−4.10		
**4**	−4.51(R+)			−4.14(S−)	−7.31	−6.45	1	**Ser115**, Tyr224
**5**	−4.96(R+)		−1.53(S−)	−4.76(R+)	−5.52	−5.14		
**6**	−3.41(R+)			−5.66(S−)	−7.10	−6.80	2	**Ser115**
**7**	−4.40(R+)	−2.33(S−)		−4.71(R+)	−5.82	−5.62		
**8**	−4.56(S−)		−1.95(S−)	−5.92(R+)	−7.80	−7.53	1	**His285**
**9**	−5.67(R+)			−6.11(R+)	−6.01	−5.16		
**10**	−3.35(S−)		−2.58(R+)	−4.22(S−)	−4.76	−4.24		
**11**	−2.14(S−)	−1.28(S−)	−2.10(S−)	−3.01(S−)	−4.16	−3.28		
**12**	−3.45(R+)			−3.61(S−)	−4.50	−3.95		
**13**	−4.55(R+)		−1.42(S−)	−3.34(R+)	−6.02	−5.12	1	Tyr138
**14**	−2.03(R+)			−1.22(S−)	−6.13	−5.10	1	Tyr224
**15**	−5.01(R+)	−3.17(R+)	−2.99(R+)	−4.86(R+)	−6.14	−6.11	1	Tyr224
**16**	−4.17(S−)	−1.09(R+)		−6.33(R+)	−8.63	−7.58	2	**Ser115**, Tyr224
**17**	−2.92(R+)			−4.18(S−)	−6.11	−5.13	1	Tyr224
**18**	−5.21(S−)	−1.78(S−)	−2.86(R+)	−6.53(R+)	−8.52	−7.64	1	**Ser115**
**19**	−4.69(R+)	−1.62(S−)		−6.71(R+)	−8.11	−7.95	3	Tyr138, **His285**, Gly286

## Data Availability

Not applicable.

## Supplementary Materials

The following are available online, Table S1: PASS prediction results.
