# Supplementary material for: Exploration of the Antimicrobial Effects of Benzothiazolylthiazolidin-4-One and In Silico Mechanistic Investigation
Source: Molecules. 2021 Jul 2;26(13):4061. doi: 10.3390/molecules26134061 (PMC8271899; doi:10.3390/molecules26134061)
Supplement: Supplementary file 1 [file molecules-26-04061-s001.zip › molecules-1255178-supplementary.pdf]

molecules

MDPI

Supplementary Materials

Exploration of the Antimicrobial Effects of Benzothiazolylthiazolidin-4-One and In Silico Mechanistic Investigation

Michelyne Haroun [1,*], Christophe Tratrat [1], Anthi Petrou [2], Athina Geronikaki [2,*], Marija Ivanov [3], Ana Ćirić [3], Marina Soković [3], Sreeharsha Nagaraja [1,4], Katharigatta Narayanaswamy Venugopala [1,5], Anroop Balachandran Nair [1], Heba S. Elsewedy [1] and Hafedh Kochkar [6,7]

[1] Department of Pharmaceutical Sciences, College of Clinical Pharmacy, King Faisal University, Al-Ahsa 31982, Saudi Arabia; christophetratrat@gmail.com (C.T.); sharsha@kfu.edu.sa (S.N.); kvenugopala@kfu.edu.sa (K.N.V.); Anair@kfu.edu.sa (A.B.N.); helsewedy@kfu.edu.sa (H.S.E.)
[2] School of Pharmacy, Aristotle University of Thessaloniki, 54124 Thessaloniki, Greece; anthi.petrou.thessaloniki1@gmail.com
[3] Mycological Laboratory, Department of Plant Physiology, Institute for Biological Research, Siniša Stanković-National Institute of Republic of Serbia, University of Belgrade, Bulevar Despota Stefana 142, 11000 Belgrade, Serbia; marija.smiljkovic@ibiss.bg.ac.rs (M.I.); rancic@ibiss.bg.ac.rs (A.Ć.); mris@ibiss.bg.ac.rs (M.S.)
[4] Department of Pharmaceutics, Vidya Siri College of Pharmacy, Off Sarjapura Road, Bengaluru 560 035, Karnataka, India
[5] Department of Biotechnology and Food Technology, Faculty of Applied Sciences, Durban University of Technology, Durban 4001, South Africa
[6] Department of Chemistry, College of Science, Imam Abdulrahman Bin Faisal University, Dammam 31441, Saudi Arabia; hbkochkar@iau.edu.sa
[7] Basic & Applied Scientific Research Center, Imam Abdulrahman Bin Faisal University, Dammam 31441, Saudi Arabia
* Correspondence: mharoun@kfu.edu.sa (M.H.); geronik@pharm.auth.gr (A.G.); Tel.: +30-2310-997-616 (A.G.)

Table S1. PASS prediction results.

No	Pa	Pi	Activity	No	Pa	Pi	Activity
**1**	0,211	0,108	Antibacterial	**13**	0,296	0,062	Antibacterial
	0,416	0,066	Muramoyltetrapeptide carboxypeptidase inhibitor		0,575	0,028	Muramoyltetrapeptide carboxypeptidase inhibitor
**2**	0,454	0,053	Muramoyltetrapeptide carboxypeptidase inhibitor	**14**	0,337	0,047	Antibacterial
**3**	0,355	0,095	Muramoyltetrapeptide carboxypeptidase inhibitor		0,624	0,022	Muramoyltetrapeptide carboxypeptidase inhibitor
**4**	0,454	0,053	Muramoyltetrapeptide carboxypeptidase inhibitor	**15**	0,224	0,099	Antibacterial
**5**	0,439	0,058	Muramoyltetrapeptide carboxypeptidase inhibitor		0,437	0,058	Muramoyltetrapeptide carboxypeptidase inhibitor
**6**	0,392	0,076	Muramoyltetrapeptide carboxypeptidase inhibitor	**16**	0,315	0,055	Antibacterial
**7**	0,314	0,125	Muramoyltetrapeptide carboxypeptidase inhibitor		0,415	0,066	Muramoyltetrapeptide carboxypeptidase inhibitor
**8**	0,400	0,073	Muramoyltetrapeptide carboxypeptidase inhibitor	**17**	0,277	0,069	Antibacterial
**9**	0,400	0,073	Muramoyltetrapeptide carboxypeptidase inhibitor		0,569	0,029	Muramoyltetrapeptide carboxypeptidase inhibitor
**10**	0,240	0,089	Antibacterial	**18**	0,238	0,090	Antibacterial
	0,451	0,054	Muramoyltetrapeptide carboxypeptidase inhibitor		0,451	0,054	Muramoyltetrapeptide carboxypeptidase inhibitor
**11**	0,330	0,049	Antibacterial	**19**	0,315	0,055	Antibacterial
	0,429	0,061	Muramoyltetrapeptide carboxypeptidase inhibitor		0,607	0,024	Muramoyltetrapeptide carboxypeptidase inhibitor
**12**	0,226	0,097	Antibacterial				
	0,451	0,054	Muramoyltetrapeptide carboxypeptidase inhibitor				
